# Effect of a valgus brace on medial tibiofemoral joint contact force in knee osteoarthritis with varus malalignment: A within-participant cross-over randomised study with an uncontrolled observational longitudinal follow-up

**DOI:** 10.1371/journal.pone.0257171

**Published:** 2022-06-03

**Authors:** Michelle Hall, Scott Starkey, Rana S. Hinman, Laura E. Diamond, Gavin K. Lenton, Gabrielle Knox, Claudio Pizzolato, David J. Saxby

**Affiliations:** 1 Centre for Health, Exercise and Sports Medicine, Department of Physiotherapy, School of Health Sciences, Melbourne, Victoria, Australia; 2 Griffith Centre of Biomedical and Rehabilitation Engineering (GCORE), Menzies Health Institute Queensland, Griffith University, Gold Coast, Queensland, Australia; Prince Sattam Bin Abdulaziz University, College of Applied Medical Sciences, SAUDI ARABIA

## Abstract

**Background:**

Previous investigations on valgus knee bracing have mostly used the external knee adduction moment. This is a critical limitation, as the external knee adduction moment does not account for muscle forces that contribute substantially to the medial tibiofemoral contact force (MTCF) during walking. The aims of this pilot study were to: 1) determine the effect of a valgus knee brace on MTCF; 2) determine whether the effect is more pronounced after 8 weeks of brace use; 3) assess the feasibility of an 8-week brace intervention.

**Methods:**

Participants with medial radiographic knee OA and varus malalignment were fitted with an Össur Unloader One^©^ brace. Participants were instructed to wear the brace for 8 weeks. The MTCF was estimated via an electromyogram-assisted neuromuscular model with and without the knee brace at week 0 and week 8. Feasibility outcomes included change in symptoms, quality of life, confidence, acceptability, adherence and adverse events.

**Results:**

Of the 30 (60% male) participants enrolled, 28 (93%) completed 8-week outcome assessments. There was a main effect of the brace (p<0.001) on peak MTCF and MTCF impulse, but no main effect for time (week 0 and week 8, p = 0.10), and no interaction between brace and time (p = 0.62). Wearing the brace during walking significantly reduced the peak MTCF (-0.05 BW 95%CI [-0.10, -0.01]) and MTCF impulse (-0.07 BW.s 95%CI [-0.09, -0.05]). Symptoms and quality of life improved by clinically relevant magnitudes over the 8-week intervention. Items relating to confidence and acceptability were rated relatively highly. Participants wore the brace on average 6 hrs per day. Seventeen participants reported 30 minor adverse events over an 8-week period.

**Conclusion:**

Although significant, reductions in the peak MTCF and MTCF while wearing the knee brace were small. No effect of time on MTCF was observed. Although there were numerous minor adverse events, feasibility outcomes were generally favourable.

**Trial registration:**

Australian and New Zealand Clinical Trials Registry (12619000622101).

## Background

Patients with medial tibiofemoral knee OA and varus malalignment have greater functional and structural decline compared to those with OA and more neutral knee alignment [1, 2]. The poorer prognosis in people with medial tibiofemoral knee OA and varus malalignment is thought to be driven by higher medial compressive loads [3, 4]. Biomechanical treatments rank highly as a research priority amongst people with OA and clinicians [5], and may be particularly applicable to people with medial tibiofemoral knee OA and varus malalignment.

Clinical guidelines for knee OA management provide conflicting recommendations for use of knee bracing. The 2019 American College of Rheumatology guidelines recommend knee bracing for tibiofemoral knee OA management [6], while the 2019 Osteoarthritis Research Society International guidelines do not recommend knee bracing for knee OA [7]. Valgus knee bracing is thought to alleviate symptoms of medial knee OA and potentially slow structural progression by reducing loads borne through the medial tibiofemoral compartment during walking. However, the effect of valgus knee bracing on medial tibiofemoral compartment compressive loads is not well understood. Low-quality evidence suggests valgus bracing creates a moderate to large reduction in the external knee adduction moment during walking [8].

Use of the external knee adduction moment to infer medial tibiofemoral joint loading is a critical limitation of the literature to date, as it does not account for the contribution of muscle forces to the medial tibiofemoral joint contact force (MTCF) [9, 10]. Muscle forces stabilise the knee in the frontal plane and account for a considerable proportion of the MTCF during walking [10, 11]. Muscular adaptations in response to wearing a knee valgus brace have been reported [12, 13], such as a reduction in co-contraction in muscles crossing the knee which can alter the MTCF. Moreover, there is little consensus to how long a patient with knee OA should wear a valgus knee brace [14] and understanding brace effects on MTCF beyond the immediate effects is necessary to elucidate the potential mechanisms of clinical effects in the longer-term.

In people with medial tibiofemoral knee OA and varus malalignment the study aims of this study were to determine: 1) the effect of valgus knee brace on MTCF (peak and impulse), including external and muscle contributions during walking; 2) whether the effects of valgus knee brace on MTCF (peak and impulse) are more pronounced after 8 weeks of brace use compared to the effect assessed at week 0; and 3) determine the feasibility of an 8-week brace intervention (changes in knee OA symptoms, quality of life, adverse events, adherence and acceptability).

## Methods

The study was registered in the Australian and New Zealand Clinical Trials Registry (12619000622101) and is reported according to the items of the CONSORT [15] applicable to pilot studies. Ethical approval was obtained from the Institutional Human Research Ethics Committee (ID: 1853473) and participants provided their written informed consent prior to testing. The study was conducted at the University of Melbourne.

### Study design

A within-participant randomised cross-over study design was used to determine effect of the valgus knee brace on MTCF (study aim 1). An observational longitudinal uncontrolled study design was used to determine: if the effect of the valgus knee brace was more pronounced at 8 weeks compared to week 0 (study aim 2), and the feasibility of an 8-week brace intervention (study aim 3). S1 Fig illustrates an overview of the study, assessment time-points and outcomes. Our report is in accordance with the Transparent Reporting of Evaluations with Nonrandomized Designs guidelines (S1 Table).

### Participants

Participants were recruited from the community in Melbourne, Australia between April 2019 and November 2019 via advertisements in social media and our volunteer database. Knee OA was classified according to the American College of Rheumatology clinical and radiographic criteria for knee OA [16]. Participants were included if they: i) were aged 50 years or older; ii) reported knee pain on most days of the past month for >3 months; iii) reported knee pain over the past week while walking of ≥4 on a numerical rating scale (NRS); iv) demonstrated radiographic tibiofemoral joint OA (Kellgren & Lawrence grade ≥2); and v) had varus malalignment [17]. Varus malalignment was defined as an anatomic axis angle of <181° for females or <183° for males [18]. Exclusion criteria were: i) lateral joint space narrowing greater than or equal to medial joint space narrowing; ii) lateral osteophyte grade greater than or equal to medial compartment osteophyte grade; iii) any knee surgery over the past 6 months; iv) awaiting or planning any back or lower-limb surgery over the next 3 months; v) planning to see an orthopaedic surgeon about knee problems over the next 8 weeks; vi) current or past (3 months) use of oral or intra-articular corticosteroid; vii) systemic arthritis; viii) current or past (6 months) muscular or joint condition other than knee OA; ix) current use of, past (6 months) use of, or intention to use (next 8 weeks) a knee brace, walking stick or gait aid; x) work restrictions or other commitment that would prevent wearing a knee brace during daily activities; and xi) unwillingness or inability to undergo magnetic resonance imaging.

### Procedures

Volunteers were screened via an online survey followed by telephone screening to confirm eligibility. Potentially eligible participants underwent a knee x-ray if they did not have their own knee x-ray within the past 12 months. For participants with bilateral symptoms, the most symptomatic eligible knee was considered as the study knee. Week 0 and week 8 participant-reported data were collected via REDCap. Participants recorded weekly data, including adverse events in a paper-based logbook. Biomechanics data were collected at the University of Melbourne by the same researcher at week 0 and week 8.

### Brace intervention

This study evaluated a valgus knee brace (Unloader One^©^, Össur, Reykjavik, Iceland, S2 Fig) and below we describe the intervention according to TIDIeR requirements [19]. For the assessment of the valgus knee brace on MTCF at week 0, participants were fitted with the knee brace by the assessor (SS) face-to-face in a one-on-one session. The fitter (SS) is a physiotherapist trained by the manufacturer in brace fitting. Brace sizing was determined by measuring the circumference of the thigh 15cm above the centre of the patella and matched to the sizing chart provided by the manufacturer. The amount of valgus force via the dynamic force straps was titrated by increasing the SmartDosing^™^ dial, until the participant reported an alleviation of their knee pain whilst walking. Participants were then asked to walk with the brace for 10 minutes prior to MTCF assessment to ensure familiarisation.

Following MTCF assessment at week 0 participants were provided with a demonstration of how to put on/take off the brace and how to self-adjust the straps, as well as written material and a video link to assist with these processes. Participants were instructed to gradually increase their brace usage by 1–2 hours per day until they were wearing the brace “whenever you are on your feet performing daily activities” for the next 8 weeks. The 8-week intervention began once the participants were fitted with their brace. In the occurrence of adverse events or incorrect issues with fitting, participants were encouraged to contact the assessor via email or phone for troubleshooting. If the problem was unable to be resolved, participants were seen face-to-face for a short refitting session at the University of Melbourne.

### Medial tibiofemoral joint contact force

#### Biomechanical data acquisition

Medial tibiofemoral joint contact was assessed when wearing the brace (i.e. braced) and without wearing a brace (i.e. unbraced) at week 0 and week 8. At each time-point, the order of assessment (i.e. braced and unbraced) was randomised to prevent any order effect. Participants walked at self-selected walking speeds matched ±5% between time-points and between braced and unbraced conditions. A full body marker set, consisting of sixty-seven reflective markers were placed on the participants skin according to a previous marker set [20], and motion tracked using a 12-camera motion analysis system (Vicon MX, Oxford Metrics, UK) at 120 Hz. Ground reaction forces were recorded using three ground-embedded force plates (AMTI, MASS, USA) at 1200 Hz. Surface electromyograms (EMG) were acquired to inform medial tibiofemoral contact forces estimates using a telemetered 16-channel wireless system (Noraxon, AZ, USA), sampling at 1200 Hz from twelve lower-limb muscles: tensor fascia latae, gluteus medius, rectus femoris, vastus lateralis, vastus medialis, biceps femoris, semimembranosus, medial gastrocnemius, lateral gastrocnemius, soleus, tibialis anterior and peroneus longus consistent with SENIAM guidelines [21]. Maximum EMG recordings for each of the twelve muscles were obtained during a set of maximum voluntary contraction (MVC) trials for the instrumented muscles: (i) seated knee extension, (ii) seated knee flexion, (iii) seated ankle eversion, (iv), seated ankle dorsiflexion, (v) standing hip abduction, and (vi) single leg heel raise. Participants performed three maximal efforts for five seconds with 30 seconds rest in between efforts.

#### Imaging acquisition and processing

A 3D T_1_-weighted sagittal vibe and a 3D T_1_-coronal scans were undertaken at week 0 using a 3-Tesla magnetic resonance imaging (MRI) machine (Siemens Medical Systems, Erlangen, Germany). Scans were acquired to inform medial tibiofemoral contact forces estimates. From these scans, three-dimensional lower limb bones and tibiofemoral joint cartilage were segmented from scans using Mimics software (Materialise, Leuven, Belgium). Bone segment dimensions, anatomical landmark coordinates, and femoral intercondylar distance were obtained using 3-Matic (Materialise, Leuven, Belgium).

#### Biomechanical modelling

Laboratory force plate, marker, and EMG data were processed within Matlab (MathWorks, 2019b) using the MOtoNMS toolbox [22]. The raw EMG data were first band-pass filtered (30–400 Hz), full-wave rectified, then low-pass filtered using a zero-lag 2^nd^ order Butterworth filter with 6 Hz low-pass frequency. The linear envelopes obtained were then amplitude-normalized to the maximum EMG value recorded during the MVC trials at respective time-points. A generic, full-body musculoskeletal model [23] was used within OpenSim [24], which had three rotational degrees of freedom at the hip, one at the knee, and one at the ankle. The hip joint centre was obtained as the centre of a sphere fitted on the respective segmented femoral head. Coordinates of key anatomical landmarks were obtained from the segmented models. Pelvis, femur, and tibia segment lengths and widths were scaled using anatomical landmark coordinates obtained from the lower limb segmentations using 3-matic (Materialise, Leuven, Belgium). Foot and torso model segment dimensions and mass properties were linearly scaled to match individual anthropometry using motion capture markers that were acquired during a static pose. The intercondylar distance (mm) was determined by conducting an extrema analysis of the most distal point between the respective femoral condyles visualized on the MRI scans. After model scaling, OpenSim inverse kinematics, inverse dynamics, and muscle analysis tools were used to determine the lower-limb joint kinematics, joint moments, and muscle-tendon unit kinematics, respectively. The brace action was modelled in OpenSim as an external load applied as a pure abduction moment about the tibia body which varied in magnitude as a function of knee flexion/extension angle, as specified by the manufacturer.

#### Neuromusculoskeletal modelling

The modelled joint moments, muscle-tendon unit kinematics, and processed EMG were then used to calibrate and then execute neuromusculoskeletal model for each participant using the Calibrated EMG-Informed Neuromusculoskeletal modelling toolbox (CEINMS) [25]. The 12 experimental EMG signals were mapped to 20 muscle-tendon units excitations in the model [25, 26]. For each participant, parameters of knee-spanning muscles were first optimized used morphometric scaling [27]. Activation dynamics and muscular model parameters were then functionally calibrated within physiological boundaries using four walking trials (one fast and normal paced trial for braced and unbraced conditions) [10, 11, 28].

Following calibration, CEINMS was used to estimate the muscle forces from experimental EMG and muscle-tendon unit kinematics for the remaining four normal paced walking trials for each condition that were not used during calibration. Muscle tendon unit dynamics were determined using assisted–mode neural solution within CEINMS was used for this dataset [26], which synthesized excitation patterns using optimization criteria for muscles that did not have experimental EMG. These muscle forces were then used as inputs into a planar knee mechanism to estimate the MTCF [11]. The relative contribution of muscle forces and external loads to compartmental tibiofemoral contact force were determined by summing the muscle moments, external torques, and contact reaction moments about the medial and lateral contact tibiofemoral points [10, 11, 28].

For each participant external loads, and tibiofemoral contact forces over each gait cycle were spline interpolated to 101 time points. The MTCF were normalised to bodyweight (BW). The absolute peak (BW) and impulse (BW.s), and the relative muscular and external loading contributions to the peak MTCF and MTCF impulse were extracted. Individual change scores (%, relative to unbraced barefoot) at each time-point for peak MTCF and MTCF impulse were also extracted. Using an EMG-driven model to estimate peak MTCF is a validated (R^2^ >0.90 [29]) and reliable (ICC 0.86 [30]) approach to estimate internal joint loads at the medial tibiofemoral compartment.

### Feasibility outcomes

Symptoms [31–33] quality of life [34], confidence performing daily tasks while wearing the brace, adverse events related to the knee brace, adherence to wearing the knee brace and acceptability of wearing the brace over 8-weeks were recorded. Table 1 summarises the instruments used to measure feasibility outcomes and time-points assessed.

**Table 1:** Outcomes.

Outcomes	Data collection instrument	Timepoints assessed		
		Week 0	Week 8	Weekly
***Study aims one and two***	Motion analysis system (Vicon MX, Oxford Metrics, UK), force plates (AMTI, MASS, USA), surface electromyography (Noraxon, AZ, USA), magnetic resonance imaging (Siemens Medical Systems, Erlangen, Germany)			
Peak medial tibiofemoral joint contact force (BW)		×	×	
External contribution to peak medial contact force (BW)		×	×	
Muscle contribution to medial contact force (BW)		×	×	
Medial tibiofemoral joint contact force impulse (BW·s)		×	×	
External contribution to medical contact force impulse (BW·s)		×	×	
Muscle contribution medical contact force impulse (BW·s)		×	×	
***Study aim three***				
Pain intensity during walking	11-point NRS (0 = no pain and 10 = worst pain possible) [31]	×	×	
Knee-related problems	KOOS questionnaire [32, 42] (each subscale 0 = extreme knee related problems and 100 = no related knee problems)	×	×	
	Pain subscale			
	Function subscale	×	×	
	Sport and recreation subscale	×	×	
	Quality of life subscale	×	×	
	Patellofemoral subscale	×	×	
Health-related quality of life	AQoL 6-D questionnaire (-0.04 = lowest quality of life and 1.00 = best quality of life [33, 34])	×	×	
Perceived change since week 0	Overall change, 7-point ordinal scale, (terminal descriptors of “much worse” to “much better” [34, 47])		×	
	Change in pain, 7-point ordinal scale (terminal descriptors of “much worse” to “much better”[34, 47])		×	
	Change in function, 7-point ordinal scale (terminal descriptors of “much worse” to “much better”[34, 47])		×	
Confidence	Self-rated confidence levels whilst performing daily tasks when wearing the brace, 11-point NRS (0 = not confident at all and 10 = extremely confident)			×
Adherence	Self-rated adherence to wearing the brace every day during daily activities, 11-point NRS (0 = have not worn brace at all and 10 = have worn brace completely as instructed)		×	
	Self-recorded daily usage of the brace (in hours)			×
Harms	Adverse events (number and nature)			×
Acceptability	Comfort levels whilst wearing the brace, 11-point NRS (0 = not at all comfortable and 10 = extremely comfortable)			×
	Ease of wearing the brace during daily life, 11-point NRS (0 = not at all easy and 10 = extremely easy)		×	
	Ease of putting the brace on and off yourself, 11-point NRS (0 = not at all easy and 10 = extremely easy)		×	
	Ease of wearing the brace with normal clothing, 11-point NRS (0 = not at all easy and 10 = extremely easy)		×	
	Likelihood of continuing to wear the brace during all daily activities in the future, after participation in this study is finished, 11-point NRS (0 = not at all likely and 10 = extremely likely)		×	
	Likelihood of recommending such a brace to a friend with similar knee problems, 11-point NRS (0 = not at all likely and 10 = extremely likely)		×	

NRS Numeric rating scale; KOOS Knee Injury Osteoarthritis Outcome Score; AQoL 6-D Assessment of Quality of Life Instrument.

### Sample size calculation

This study was powered on primary objective, where we wished to detect a small to medium bracing effect size of 0.35 for peak MTCF. Assuming 80% power, an alpha of 0.05, and a correlation between measurements on the same individual of 0.82 [9], a sample of at least 26 participants was required. To allow for 15% dropout or loss of data, we aimed to recruit 30 participants.

### Statistical analysis for study aim one and study aim two

There were two independent variables (CONDITION and TIME). The two levels of CONDITION were brace and no brace, and the two levels of TIME were week 0 and week 8. Dependent variables included 1) peak MTCF; 2) MTCF impulse; 3) external component of the peak MTCF; 4) muscle component of the peak MTCF; 5) external component of the MTCF impulse; 6) the muscle component of the MTCF impulse and 7) walking speed. A repeated-measures multivariate analysis of variance (MANOVA) was performed to evaluate the main effects and interaction of the independent variables on the dependent variables collectively, thereby controlling for experiment-wise error rate. Assumptions including homogeneity of variances of the residuals, normal distribution of the residuals and independence observations were evaluated. In the event of a significant main or interaction effect, univariate analysis of variance was performed to explore significant effects.

### Statistical analysis for study aim three

For knee OA symptoms and quality of life, data for participants who had data at week 0 and week 8 were used to calculate change scores (week 8 minus week 0) with 95% confidence intervals for continuous scores. The number of participants (percentage) who reached minimal clinically important difference in NRS knee pain intensity during level walking (at least 1.8 units [31]), in The Knee injury and Osteoarthritis Outcome Score subscales (at least 10 units [35] for pain, function in activities of daily living, function in sport and recreation and knee-related quality of life; at least 14 units [36] for the patellofemoral subscale) and the Assessment of Quality of Life 6-D (at least 0.6 units [37]). Means, standard deviations and average ranges (i.e. the average of the minimum values and average of the maximum values) were used to describe weekly confidence, adherence and comfort data. Categorical data were expressed as number of participants (percentage). All statistical analyses were performed using Statistical Package for Social Sciences (SPSS), version 25 (IBM, New York, USA) with significance at p < 0.05.

## Results

**Fig 1:**
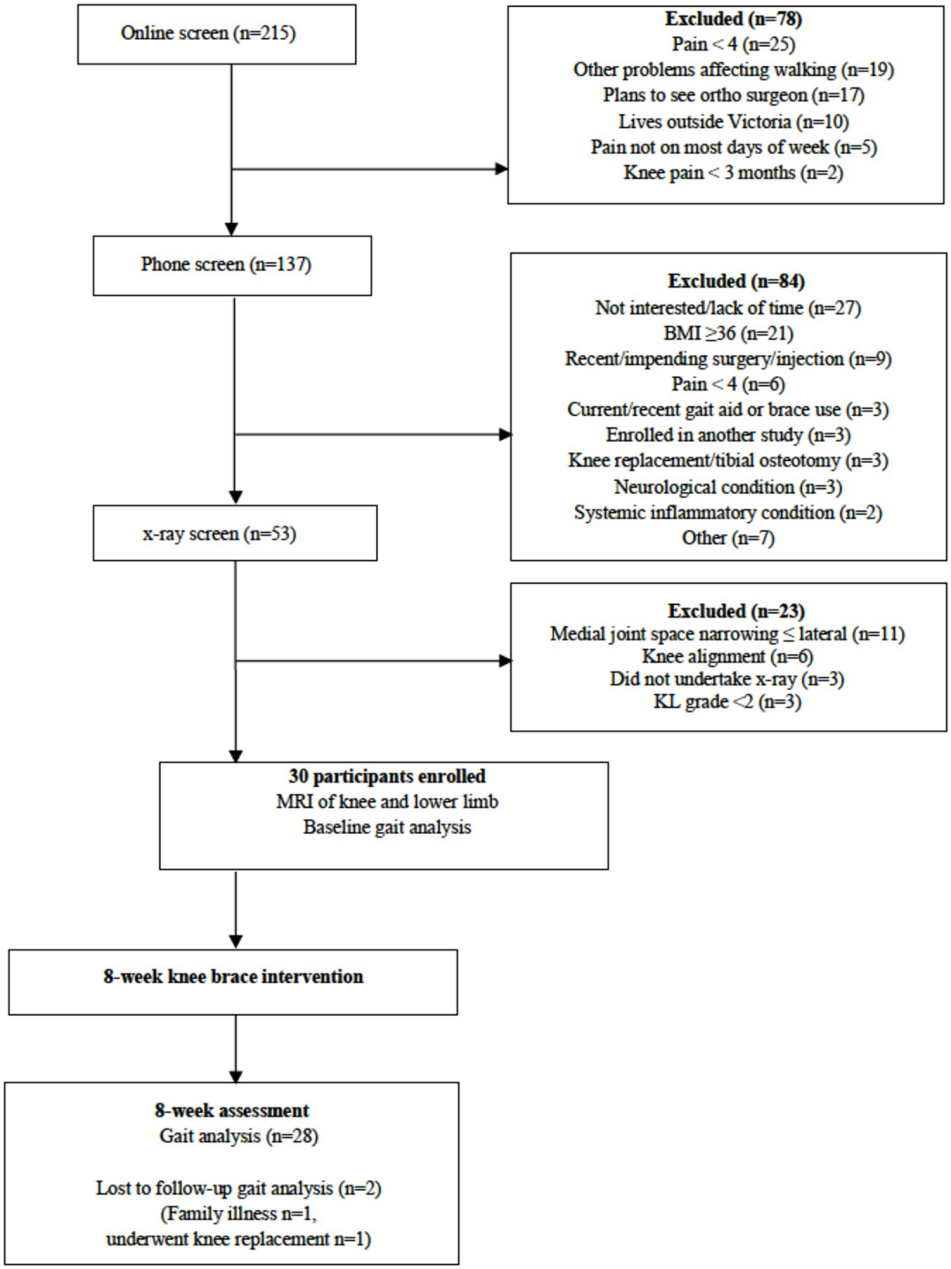
Flow of participants through the study.

Of the 211 individuals who completed initial online screening, 133 (63%) passed phone screening, 47 (22%) passed x-ray screening, 33 (16%) passed knee alignment assessment and 30 (14%) participants fulfilled eligibility criteria and were enrolled into the study (Fig 1). Twenty-eight (93%) of the 30 participants enrolled completed week 8 MTCF assessment. One participant relocated interstate while the second underwent an unplanned total knee replacement. The cohort had slightly more males, were overweight, and had predominantly moderate-to-severe radiographic knee OA (Table 2).

**Table 2:** Participant characteristics.

	n = 30
Age, yr	64.1 (4.7)
Male, n (%)	18 (60%)
Height, m	1.69 (0.10)
Weight, kg	85.0 (13.7)
Body mass index, kg/m^2^	29.7 (3.3)
Unilateral symptoms, n (%)	16 (53%)
Duration of symptoms, yr	5.2 (4.5)
Average pain over the past week^a^	6.14 (1.56)
Most affected leg, righ t(%)	23 (77%)
Test leg dominant, yes (%)	26 (87%)
Knee alignment^b^, degrees	
Females	178.2 (2.6)
Males	177.9 (3.1)
Radiographic disease severity grade^c^, n (%)	
Grade 2	9 (30%)
Grade 3	12 (40%)
Grade 4	9 (30%)

Except where indicated otherwise, values are the mean (SD);

^a^Numeric rating (0 = no pain and 10 = worst pain possible);

^b^Anatomic alignment, where neutral alignment is 181° for females and 183° for males and varus is <181° for females and <183° for males;

^c^Kellgren-Lawrence grading system.

### Study aim 1) Determine the effect of valgus knee brace on MTCF (peak and impulse), including external and muscle components during walking

Discrete measures and waveforms of the peak MTCF and MTCF impulse along with the respective external and muscle contributions are described in Table 3 and illustrated in S3 Fig), respectively. The results of the repeated measures MANOVA showed a significant effect of CONDITION (p<0.001). Subsequent univariate analysis showed a significant effect of the brace on the peak MTCF (p = 0.016) and the MTCF impulse (p<0.001). Wearing the brace during walking reduced the peak MTCF (-0.05 BW 95%CI [-0.10, -0.01]) and MTCF impulse (-0.07 BW.s 95%CI [-0.09, -0.05]) compared to walking without the brace. There was a significant effect of CONDITION on the external and muscle components of the peak MTCF (external p<0.001; muscle p = 0.04) and MTCF impulse (external p<0.001 and muscle p<0.01). For the peak MTCF, the external component was lower (-0.09 BW 95%CI [-0.13, -0.05]) and the muscle component was higher (0.03 BW 95%CI [0.00, 0.07]) walking with the brace compared to walking without the brace. Both the external (-0.05 BW.s 95%CI [-0.07, -0.03]) and muscle (-0.02 BW.s 95%CI [-0.03, -0.01]) components of the MTCF impulse was lower walking with the brace compared to walking without the brace. The effect of CONDITION on walking speed was not statistically significant (p = 0.09).

**Table 3:** Spatiotemporal and joint contact force related variables for brace and no brace conditions at week 0 and week 8.

	Week 0			Week 8		
	Brace (n = 30)	No Brace (n = 30)	Mean difference (95% CI)	Brace (n = 28)	No Brace (n = 28)	Mean difference (95% CI)
Walking speed (m/s)	1.23 ± 0.18	1.21 ± 0.22	0.02 (-0.02, 0.06)	1.28 ± 0.18	1.25 ± 0.21	0.03 (0.01, 0.06)
Peak medial tibiofemoral joint contact force (BW)	1.92 ± 0.39	1.97 ± 0.41	-0.06 (-0.11, 0.00)	1.95 ± 0.34	2.01 ± 0.39	**-0.06 (-0.12, -0.01)**
External contribution to peak medial contact force (BW)	1.06 ± 0.40	1.15 ± 0.43	**-0.08 (-0.12, -0.04)**	1.00 ± 0.41	1.09 ± 0.44	**-0.10 (-0.14, -0.06)**
Muscle contribution to medial contact force (BW)	0.85 ± 0.27	0.83 ± 0.27	0.02 (-0.02, 0.07)	0.95 ± 0.29	0.92 ± 0.27	0.03 (-0.02, 0.08)
Medial tibiofemoral joint contact force impulse (BW·s)	0.79 ± 0.18	0.85 ± 0.21	**-0.06 (-0.09, -0.03)**	0.76 ± 0.15	0.85 ± 0.19	**-0.08 (-0.11, -0.06)**
External contribution to medical contact force impulse (BW·s)	0.46 ± 0.17	0.51 ± 0.19	**-0.05 (-0.07, -0.03)**	0.41 ± 0.19	0.38 ± 0.12	**-0.06 (-0.08, -0.04)**
Muscle contribution medical contact force impulse (BW·s)	0.33 ± 0.12	0.34 ± 0.13	**-0.01 (-0.03, 0.01)**	0.35 ± 0.11	0.47 ± 0.21	**-0.02 (-0.04, -0.01)**

Values are mean ± standard deviation; BW = body weight; CI = confidence interval; Bold indicates that confidence interval does not include zero.

### Study aim 2) Determine whether the effect of the valgus knee brace on MTCF (peak and impulse) is more pronounced after 8 weeks of brace wear

**Fig 2:**
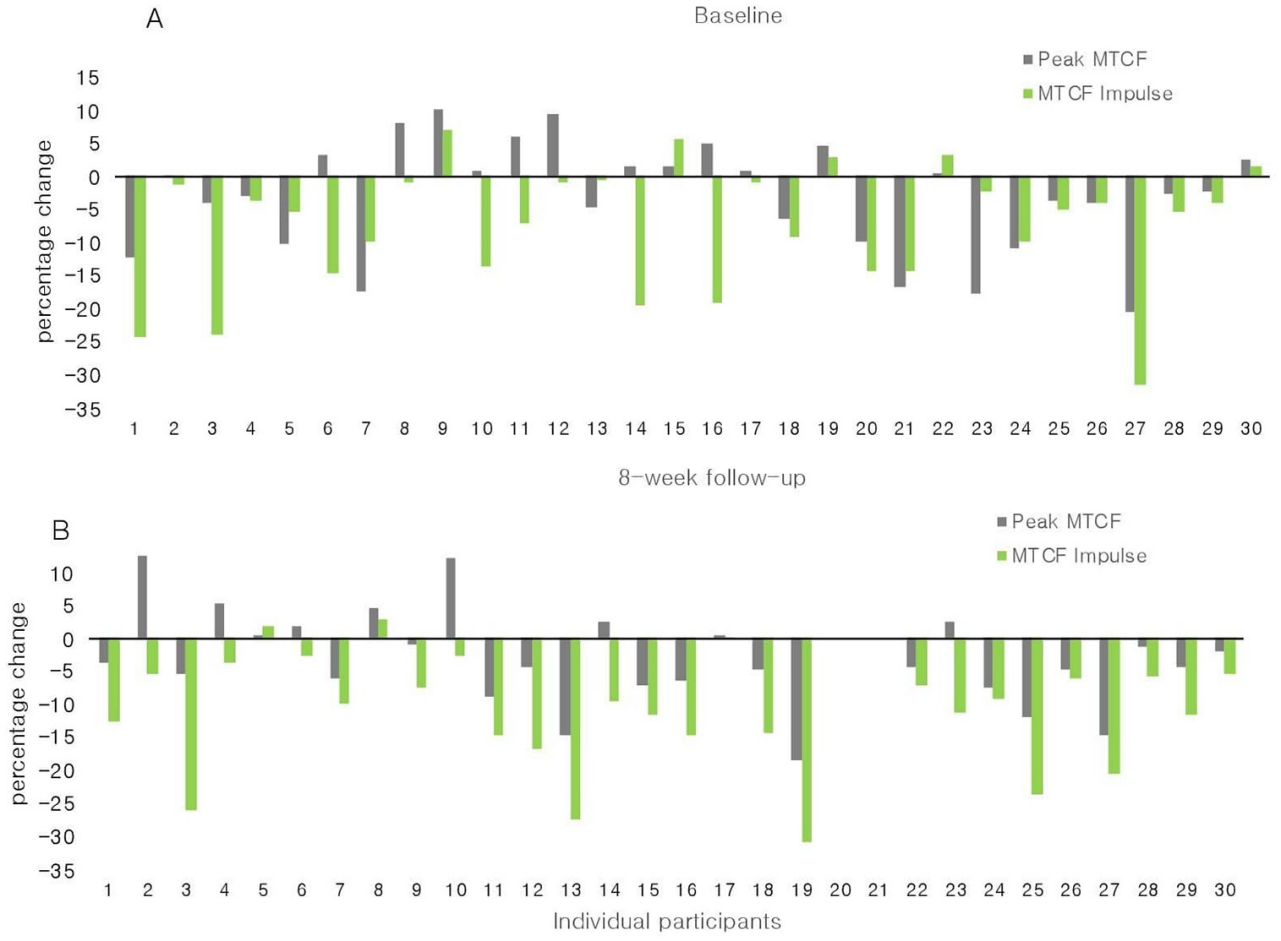
Percentage changes scores for individual participants for peak medial tibiofemoral joint contact force (MTCF) and MTCF impulse at week 0 (A) and week 8 (B). Negative values indicate a decrease in scores with wearing the brace, and positive values indicate an increase in score with wearing the brace. Missing data from two participants who did not return for MTCF assessment at week 8.

The CONDITION x TIME interaction (p = 0.62) was not statistically significant, and therefore not considered in further analyses. Removing the non-significant CONDITION x TIME interaction term from the model did not change results of the main effect. Upon visual inspection of individual data (Fig 2), there was notable inter-participant variation in the magnitude of the peak MTCF between CONDITION and TIME.

### Study aim 3) Feasibility of the 8-week brace intervention

**Fig 3:**
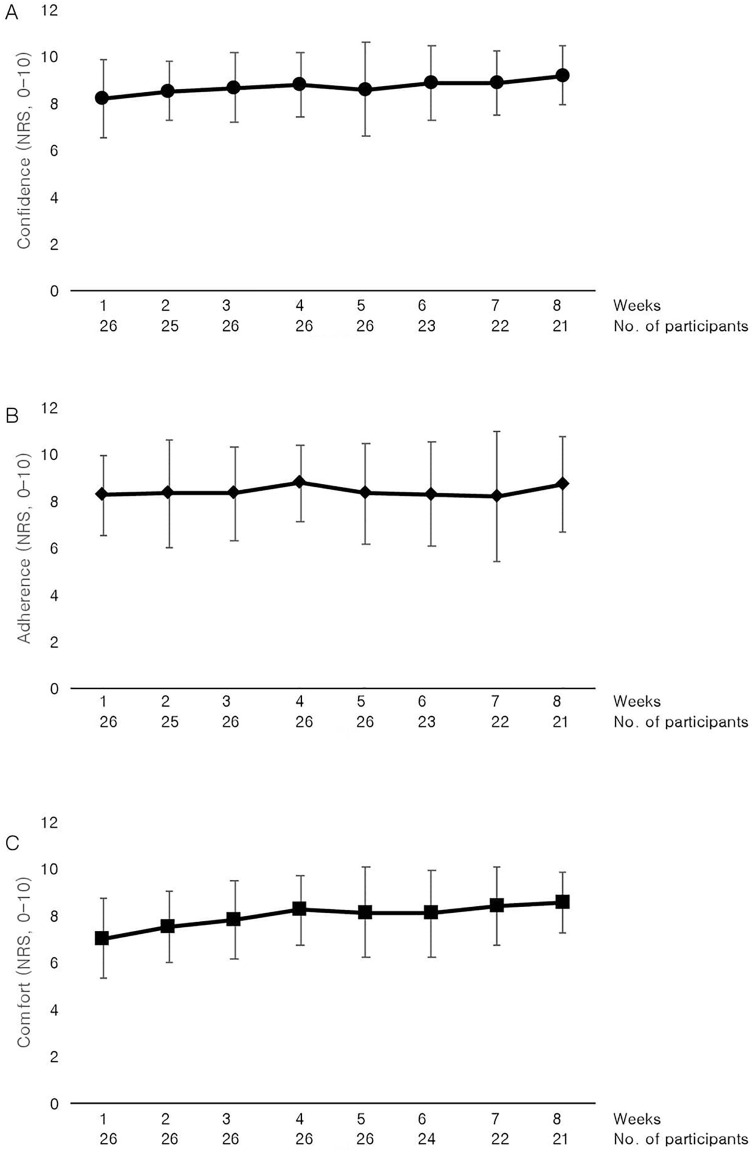
Mean and standard deviation weekly numeric rating scale (NRS) scores for confidence (A), adherence (B) and comfort (C). Higher scores indicate greater confidence levels whilst performing daily tasks when wearing the brace (A), greater adherence to wearing the brace every day during daily activities (B), greater comfort when wearing the brace; and vice versa for lower scores.

Symptoms and quality of life improved on the group level, with many participants reaching minimal clinical important differences at week 8 compared to week 0 (Table 4). On the global rating scales, 18 (64%) participants “improved” (i.e. reported “moderately better” or “much better”) their pain, and 15 (54%) participants “improved” overall and their physical function (see S4 Fig). The weekly confidence level on the 11-point NRS while performing daily tasks when wearing the brace was mean (SD), [average range] 8.7 (0.3), [8.2 to 9.2] (Fig 3a). Participants reported wearing the brace a mean (SD), [range] hours per day: 6 (3) [1–11], see S5 Fig for weekly report of the number of hours worn per week. The weekly adherence levels on the 11-point NRS to wearing the knee brace as instructed was mean (SD), [average range] 8.4 (0.2), [8.2 to 8.8] (Fig 3b). Participant acceptability of the brace was relatively high (S6 Fig), including weekly comfort levels on the 11-point NRS while wearing the brace with mean (SD), [average range] 8.0 (0.5), [7.0 to 8.6] (Fig 3c). During the intervention participants contacted the research team three times, twice for advice on skin irritation and one for brace-fitting. Skin irritation queries were discussed over the phone while one participant received face-to-face brace-refitting. Seventeen participants reported 30 minor adverse events (Table 5), all of which were considered relatively minor in nature.

**Table 4:** Patient reported outcomes. Mean ± SD at week 0 and week 8 with mean difference (week 8 minus week 0) and 95% confidence intervals (CI).

Outcome	Week 0 (n = 30)	Week 8 (n = 29)	Mean Difference (95% CI)	Number (%) of participants who met or exceed MCID**
Pain during walking (NRS)^a^*	6.1 ± 1.6	2.8 ± 1.9	-3.3 (-4.1, 2.6)	25 (89%)
KOOS^b^				
Pain	49.2 ± 14.7	69.6 ± 14.2	20.4 (14.9 25.8)	22 (76%)
Function	57.4 ± 20.4	77.5 ± 17.2	20.5 (14.2, 26.8)	22 (76%)
Sport and recreation	24.2 ± 19.1	47.8 ± 28.8	22.9 (14.6, 31.3)	22 (76%)
Quality of life	28.1 ± 15.6	46.6 ± 19.8	18.5 (10.9, 26.2)	17 (59%)
Patellofemoral	27.6 ± 15.7	53.8 ± 26.6	25.9 (18.1, 33.6)	18 (62%)
AQoL 6-D^c^	0.70 ± 0.20	0.78 ± 0.19	0.07 (0.03, 0.12)	15 (52%)

^a^ Numeric Rating Scale–Scored from 0 (no pain) to 10 (worst pain imaginable).

*data available for 28 participants at week 8.

^b^ Knee osteoarthritis outcome score (KOOS). 0 = extreme knee related problems and 100 = no related knee problems

^c^ Assessment of Quality of Life Instrument (-0.04 = lowest quality of life and 1.00 = best quality of life)

**Minimal clinical important difference.

**Table 5:** Adverse events, n (%).

Total number of adverse events	n = 30
Skin irritation	11 (37%)
Increased study knee pain	5 (25%)
Contralateral knee/hip pain	3 (10%)
Back pain	1 (3%)
Pain in other area	1 (3%)

## Discussion

The effects of valgus knee bracing on the MTCF during walking in people with knee OA and varus malalignment, are unknown. This is important to understand given reduction in the MTCF load is the premise by which valgus knee bracing is thought to have clinical benefit. In this study, the valgus knee brace reduced the MTCF, but effects were not more pronounced after 8 weeks. The mechanisms by which the MTCF reduced with the valgus brace appear to relate to a reduction in the contribution from external loads, with inconsistent changes in the contributions from knee spanning muscle to the peak MTCF and MTCF impulse. Although there were numerous adverse events, feasibility outcomes were generally favourable.

The peak MTCF and MTCF impulse reduced on average by approximately 3% and 8%, respectively, with the knee brace. These magnitudes are considerably smaller than reductions in *in vivo* medial tibiofemoral compartment load (~25%) from three individuals wearing a knee brace assessed in a previous study [38]. The majority of participants reduced MTCF measures, particularly MTCF impulse (Fig 2), albeit the minimal detectable change in MTCF impulse is unknown. Recent research has demonstrated an increase in medial compartment joint space distance in response to wearing a knee brace in all participants (n = 20) assessed [39]. However, somewhat consistent with MTCF observations in the current study with respect to magnitudes of MTCF, the individual change in joint space was variable. Knee pain during walking improved by a clinically relevant amount in 89% of our participants at week 8. It is unclear how much of the improvements in symptoms and knee-related problems are related to contextual effects [40], and whether the pain relief reported with wearing the knee brace is related to reduced mechanical load and/or improved joint health. In an uncontrolled study of 16 people with medial knee OA and varus malalignment, the number of bone marrow lesions reduced after 12 weeks of wearing the Össur Unloader One knee brace [41], which may contribute to reduced knee pain. In a clinical trial, a 6-week patellofemoral brace intervention improved bone marrow lesions [42], but these improvements did not correlate with pain relief. Understanding whether the reduction in MTCF mediates knee pain relief and/or improves knee joint health is necessary to elucidate a minimal clinical important change in MTCF required for therapeutic interventions to target.

The MTCF reductions were not more pronounced at week 8 compared to week 0, despite excellent self-reported adherence to wearing the knee brace (~6 hrs per day). Based on previous research [13], we anticipated that muscular adaptations such as a reduction in co-contraction of the knee muscles would occur over time and further lower the MTCF. Although muscle co-contractions were not assessed in this study, muscle forces that contribute to the MTCF, the metrics of interest, were assessed. We observed no effect of treatment time on muscle force contributions to the MTCF. One consideration is that 8-weeks was too short to observe neuromuscular adaptations, however other research has demonstrated reduced co-contraction within two weeks of brace wear [13]. Walking speed was controlled between the braced and unbraced conditions, but not across time-points as we anticipated a change in walking speed due to changes in pain. Nevertheless, this result remained unchanged when adjusting for walking speed as a covariate. Our findings are indirectly similar to research where the immediate effects of brace wearing on surrogate measures of tibiofemoral contact force (the external knee adduction moment) were no more pronounced after 2 weeks [43], 5 weeks [44] and 3 months [45] of wearing the brace. However, we have extended previous literature by demonstrating that muscular contribution to the MTCF does not adapt in response to wearing a knee valgus brace over 8-weeks.

Our study is unique due to our application of an EMG-assisted neuromusculoskeletal model, including assessments of the external and muscle contributions to the MTCF. Muscle forces act to stabilize the knee against external loads and account for a considerable proportion (>50%) of the MTCF [10, 11]. The external component of both the peak MTCF and MTCF impulse reduced in the braced condition compared to the unbraced condition. This is logical given that a valgus torque was applied via the brace in people who had varus malalignment. Interestingly, the contribution of the muscle component increased for the peak MTCF and decreased for MTCF impulse. The conflicting observations for contribution of the muscle component for the MTCF are unclear. It should be noted however, that despite the increase in the absolute muscle contribution to peak MTCF, the peak MTCF reduced with wearing the brace. The external and muscle components that contribute to the MTCF can be modulated through various coordination strategies [46], that are individual-specific and challenging to disentangle. Investigations into subgroups using clustering techniques may provide insight into strategies adapted by individuals with knee OA when walking with a knee brace.

Improvements in symptoms and, to a lesser extent quality of life, were clinically relevant for many participants. However, no inferences should be made using symptom data given the lack of a control group. Adverse events are generally poorly reported in brace studies [8], but the nature of our adverse events are consistent with those synthesised in a review [8]. Nevertheless, the number of adverse events is higher (n = 30) than a 12-month brace intervention in 60 participants [47] (n = 24) and modifications to our brace intervention protocol used in this study may be required in a future clinical trial. For example, some adverse events may be preventable (e.g. skin irritation), by providing more regular professional re-fitting. However, additional appointments may over burden participants. Fundamental to treatment success is patient acceptability. Participants reported the brace was relatively easy to use and indicated they would continue to wear the brace upon completion of the 8-week study, indirectly suggesting that extending treatment duration would be acceptable to evaluate longer-term clinical effects.

Limitations of the study warrant consideration. First, validation of EMG-assisted NMS models is hindered by limited datasets to directly validate tibiofemoral contact force predictions [48]. Second, we did not have a sham brace condition. Third, our study sample included more males, and knee OA affects more women than men [49]. Lastly, our findings are only generalisable to the those with varus malalignment and the intervention evaluated, including the duration and the brace evaluated.

## Conclusions

Our findings indicate that valgus knee bracing in people with medial tibiofemoral knee OA and varus malalignment reduces the MTCF during walking by small amounts. Effects of the valgus knee brace on the MTCF were not more pronounced after 8 weeks of wearing the valgus knee brace. Despite favourable improvements in symptoms and quality of life, the small changes in peak MTCF and MTCF impulse magnitude questions whether MTCF reduction is the driver of symptom improvement. Our observations can be used to further refine use of a valgus knee brace for knee OA management.

## Supporting information

S1 Fig Study overview. Outcomes and time-points of assessment according to each study aim.
(PDF)

S2 Fig Unloader one^®^ (Össur, Reykjavik, Iceland). (PDF)

S3 Fig Medial tibiofemoral joint contact force. Ensemble average (± standard deviation) of the peak medial tibiofemoral joint contact force (MTCF) and MTCF impulse with the valgus knee brace (red line) and without the valgus knee brace (grey line), along with external and muscle components over a gait cycle at baseline and 8-weeks follow-up.
(PDF)

S4 Fig Global rating scores for overall change, and change in function and pain. The numbers within the horizontal bars represent the number of participants selecting each option (i.e. slight worse, no change, slightly better, moderately better and much better). One participant who underwent unplanned total knee replacement did not complete these items.
(PDF)

S5 Fig Hours brace worn. Weekly report of the number of hours the valgus knee brace was worn per week. The number of participants providing weekly data are described, some participants did not provide hours each week for various reasons.
(PDF)

S6 Fig Numeric rating scale (NRS) scores for acceptabiltiy realated questions. Higher NRS scores indicating more acceptability (10 = extremely easy/likely) and lower scores indicating less acceptabiltiy (0 = not at all easy/likely). Twenty-eight participants responded to these questions at 8-week follow-up. One participant who underwent unplanned total knee replacement did not complete these questions and another participants did not complete these items on the questionairre.
(PDF)

S1 Table TREND statement checklist. (DOCX)

S1 File Protocol. (PDF)

S2 File Study protocol—Ethics. (PDF)

S1 Database (XLSX)

S1 Checklist TREND statement checklist. (PDF)

## References

[1] Sharma L, Song J, Dunlop D, Felson D, Lewis CE, Segal N, et al. Varus and valgus alignment and incident and progressive knee osteoarthritis. Ann Rheum Dis 2010; 69:1940–1945. doi: 10.1136/ard.2010.129742 20511608 PMC2994600

[2] Sharma L, Song J, Felson DT, Cahue S, Shamiyeh E, Dunlop DD. The role of knee alignment in disease progression and functional decline in knee osteoarthritis. JAMA 2001;286:188–195. doi: 10.1001/jama.286.2.188 11448282

[3] Dell’Isola A, Smith SL, Andersen MS, Steultjens M. Knee internal contact force in a varus malaligned phenotype in knee osteoarthritis. Osteoarthritis Cartilage 2017; 25:2007–2013. doi: 10.1016/j.joca.2017.08.010 28882753

[4] Foroughi N, Smith R, Vanwanseele B. The association of external knee adduction moment with biomechanical variables in osteoarthritis: a systematic review. Knee 2009;16:303–309. doi: 10.1016/j.knee.2008.12.007 19321348

[5] Kolasinski SL, Neogi T, Hochberg MC, Oatis C, Guyatt G, Block J, et al. 2019 American College of Rheumatology/Arthritis Foundation Guideline for the management of osteoarthritis of the hand, hip, and knee. Arthritis Care Research 2020;72:149–162. doi: 10.1002/acr.24131 31908149 PMC11488261

[6] Gierisch JM, Myers ER, Schmit KM, et al. Prioritization of patient-centered comparative effectiveness research for osteoarthritis. Ann Intern Med 2014;160:836–841. doi: 10.7326/M14-0318 24821227

[7] Bannuru RR, Osani MC, Vaysbrot EE, et al. OARSI guidelines for the non-surgical management of knee, hip, and polyarticular osteoarthritis. Osteoarthritis Cartilage 2019; 27:1578–1589. doi: 10.1016/j.joca.2019.06.011 31278997

[8] Moyer RF, Birmingham TB, Bryant DM, Giffin JR, Marriott KA, Leitch KM. Biomechanical effects of valgus knee bracing: a systematic review and meta-analysis. Osteoarthritis Cartilage 2015;23:178–188. doi: 10.1016/j.joca.2014.11.018 25447975

[9] Hall M, Diamond LE, Lenton GK, Pizzolato C, Saxby DJ. Immediate effects of valgus knee bracing on tibiofemoral contact forces and knee muscle forces. Gait Posture 2019;68:55–62. doi: 10.1016/j.gaitpost.2018.11.009 30458429

[10] Saxby DJ, Modenese L, Bryant AL, Gerus P, Killen B, Fortin K, et al. Tibiofemoral contact forces during walking, running and sidestepping. Gait Posture 2016;49:78–85. doi: 10.1016/j.gaitpost.2016.06.014 27391249

[11] Winby CR, Lloyd DG, Besier TF, Kirk TB. Muscle and external load contribution to knee joint contact loads during normal gait. J Biomech 2009;42:2294–2300. doi: 10.1016/j.jbiomech.2009.06.019 19647257

[12] Fantini Pagani CH, Willwacher S, Kleis B, Brüggemann GP. Influence of a valgus knee brace on muscle activation and co-contraction in patients with medial knee osteoarthritis. J Electromyogr Kinesiol 2013;23:490–500. doi: 10.1016/j.jelekin.2012.10.007 23142529

[13] Ramsey DK, Briem K, Axe MJ, Snyder-Mackler L. A mechanical theory for the effectiveness of bracing for medial compartment osteoarthritis of the knee. J Bone Joint Surg Am 2007;89:2398–2407. doi: 10.2106/JBJS.F.01136 17974881 PMC3217466

[14] Coudeyre E, Nguyen C, Chabaud A, Pereira B, Beaudreuil J, Coudreuse JM, et al. A decision-making tool to prescribe knee orthoses in daily practice for patients with osteoarthritis. Ann Phys Rehabil Med 2018;61:92–98. doi: 10.1016/j.rehab.2018.01.001 29406129

[15] Eldridge SM, Chan CL, Campbell MJ, Bond CM, Hopewell S, Thabane L, et al. CONSORT 2010 statement: extension to randomised pilot and feasibility trials. BMJ 2016; 355:i5239. doi: 10.1136/bmj.i5239 27777223 PMC5076380

[16] Altman R, Asch E, Bloch D, Bole G, Borenstein D, Brandt K, et al. Development of criteria for the classification and reporting of osteoarthritis. Classification of osteoarthritis of the knee. Diagnostic and Therapeutic Criteria Committee of the American Rheumatism Association. Arthritis Rheum 1986; 29:1039–1049. doi: 10.1002/art.1780290816 3741515

[17] Kellgren JH, Lawrence JS. Radiological assessment of osteo-arthrosis. Ann Rheum Dis 1957;16:494–502. doi: 10.1136/ard.16.4.494 13498604 PMC1006995

[18] Kraus VB, Vail TP, Worrell T, McDaniel G. A comparative assessment of alignment angle of the knee by radiographic and physical examination methods. Arthritis Rheum 2005;52:1730–1735. doi: 10.1002/art.21100 15934069

[19] Hoffmann TC, Glasziou PP, Boutron I, Milne R, Perera R, Moher D, et al. Better reporting of interventions: template for intervention description and replication (TIDieR) checklist and guide. BMJ 2014;348:g1687. doi: 10.1136/bmj.g1687 24609605

[20] Besier TF, Sturnieks DL, Alderson JA, Lloyd DG. Repeatability of gait data using a functional hip joint centre and a mean helical knee axis. J Biomech 2003; 36:1159–1168. doi: 10.1016/s0021-9290(03)00087-3 12831742

[21] Hermens HJ, Freriks B, Merletti R, Stegeman D, Blok J, Rau G, et al. European recommendations for surface electromyography. Roessingh Research and Development 1999; 8: 13–54.

[22] Mantoan A, Pizzolato C, Sartori M, Sawacha Z, Cobelli C, Reggiani M. MOtoNMS: A MATLAB toolbox to process motion data for neuromusculoskeletal modeling and simulation. Source Code Biol Med 2015;10:12. doi: 10.1186/s13029-015-0044-4 26579208 PMC4647340

[23] Rajagopal A, Dembia CL, DeMers MS, Delp DD, Hicks JL, Delp SL. Full-Body musculoskeletal model for muscle-driven simulation of human gait. IEEE Trans Biomed Eng 2016;63:2068–2079. doi: 10.1109/TBME.2016.2586891 27392337 PMC5507211

[24] Delp SL, Anderson FC, Arnold AS, Loan P, Habib A, John CT, et al. OpenSim: open-source software to create and analyze dynamic simulations of movement. IEEE Trans Biomed Eng 2007;54:1940–1950. doi: 10.1109/TBME.2007.901024 18018689

[25] Pizzolato C, Lloyd DG, Sartori M, Ceseracciu E, Besier TF, Fregly BJ, et al. CEINMS: A toolbox to investigate the influence of different neural control solutions on the prediction of muscle excitation and joint moments during dynamic motor tasks. J Biomech 2015;48:3929–3936. doi: 10.1016/j.jbiomech.2015.09.021 26522621 PMC4655131

[26] Sartori M, Farina D, Lloyd DG Hybrid neuromusculoskeletal modeling to best track joint moments using a balance between muscle excitations derived from electromyograms and optimization. J Biomech 2014; 47:3613–3621. doi: 10.1016/j.jbiomech.2014.10.009 25458151

[27] Modenese L, Ceseracciu E, Reggiani M, Lloyd DG Estimation of musculotendon parameters for scaled and subject specific musculoskeletal models using an optimization technique. J Biomech 2016; 49:141–148. doi: 10.1016/j.jbiomech.2015.11.006 26776930

[28] Killen BA, Saxby DJ, Fortin K, Gardiner BS, Wrigley TV, Bryant AL, et al. Individual muscle contributions to tibiofemoral compressive articular loading during walking, running and sidestepping. J Biomech 2018;80:23–31. doi: 10.1016/j.jbiomech.2018.08.022 30166223

[29] Manal K, Buchanan TS. An electromyogram-driven musculoskeletal model of the knee to predict *in vivo* joint contact forces during normal and novel gait patterns. J Biomech Eng 2013;135:02104 doi: 10.1115/1.4023457 23445059 PMC3705826

[30] Gardinier ES, Manal K, Buchanan TS, Snyder-Mackler L. Minimum detectable change for knee joint contact force estimates using an EMG-driven model. Gait Posture 2013; 38:1051–1053. doi: 10.1016/j.gaitpost.2013.03.014 23601782 PMC3795951

[31] Bellamy N, Carette S, Ford PM, Kean WF, le Riche NG, Lussier A, et al. Osteoarthritis antirheumatic drug trials. II. Tables for calculating sample size for clinical trials. J Rheumatol 1992;19:444–450. 1578461

[32] Roos EM, Toksvig-Larsen S. Knee injury and Osteoarthritis Outcome Score (KOOS)—validation and comparison to the WOMAC in total knee replacement. Health Qual Life Outcomes 2003;1:17. doi: 10.1186/1477-7525-1-17 12801417 PMC161802

[33] ten Klooster PM, Drossaers-Bakker KW, Taal E, van de Laar MA. Patient-perceived satisfactory improvement (PPSI): interpreting meaningful change in pain from the patient’s perspective. Pain 2006;121:151–157. doi: 10.1016/j.pain.2005.12.021 16472915

[34] Osborne RH, Hawthorne G, Lew EA, Gray LC. Quality of life assessment in the community-dwelling elderly: validation of the Assessment of Quality of Life (AQoL) Instrument and comparison with the SF-36. J Clin Epidemiol 2003;56:138–147. doi: 10.1016/s0895-4356(02)00601-7 12654408

[35] Roos EM, Lohmander LS. The Knee injury and Osteoarthritis Outcome Score (KOOS): from joint injury to osteoarthritis. Health Qual Life Outcomes 2003;1:64–64 doi: 10.1186/1477-7525-1-64 14613558 PMC280702

[36] Crossley KM, Macri EM, Cowan SM, Collins NJ, Roos EM The patellofemoral pain and osteoarthritis subscale of the KOOS (KOOS-PF): development and validation using the COSMIN checklist. British Journal of Sports Medicine 2018; 52:1130–1136. doi: 10.1136/bjsports-2016-096776 28258176

[37] Hawthorne G, Osborne R Population norms and meaningful differences for the Assessment of Quality of Life (AQoL) measure. Aust N Z J Public Health 2005; 29:136–142. doi: 10.1111/j.1467-842x.2005.tb00063.x 15915617

[38] Kutzner I, Küther S, Heinlein B, Dymke J, Bender A, Halder AM, et al The effect of valgus braces on medial compartment load of the knee joint–in vivo load measurements in three subjects. J Biomech 2011;44:1354–1360. doi: 10.1016/j.jbiomech.2011.01.014 21288522

[39] Dessinger GM, LaCour MT, Dennis DA, Kleeman-Forsthuber LT, Komistek RD Can an OA knee brace effectively offload the medial condyle? an in vivo fluoroscopic study. J Arthroplasty 2021;36:1455–1461. doi: 10.1016/j.arth.2020.10.044 33223413

[40] Zhang W, Doherty M Efficacy paradox and proportional contextual effect. Clin Immunol 2018;186:82–86. doi: 10.1016/j.clim.2017.07.018 28736278

[41] Robbins SR, Melo LRS, Urban H, Deveza LA, Asher R, Johnson VL, et al. Effectiveness of stepped-care intervention in overweight and obese patients with medial tibiofemoral osteoarthritis: a randomized controlled trial Arthritis Care Res (Hoboken) 2021;74:520–530. doi: 10.1002/acr.24148 31961489

[42] Callaghan MJ, Parkes MJ, Hutchinson CE, Gait AD, Forsythe LM, Marjanovic EJ, et al. A randomised trial of a brace for patellofemoral osteoarthritis targeting knee pain and bone marrow lesions. Ann Rheum Dis 2015;74:1164–1170. doi: 10.1136/annrheumdis-2014-206376 25596158 PMC4771926

[43] Jones RK, Nester CJ, Richards JD, Kim WY, Johnson DS, Jari S, et al. A comparison of the biomechanical effects of valgus knee braces and lateral wedged insoles in patients with knee osteoarthritis. Gait Posture 2013; 37:368–372. doi: 10.1016/j.gaitpost.2012.08.002 22920242

[44] Laroche D, Morisset C, Fortunet C, Gremeaux V, Maillefert J-F, Ornetti P Biomechanical effectiveness of a distraction–rotation knee brace in medial knee osteoarthritis: Preliminary results. Knee 2014; 21:710–716. doi: 10.1016/j.knee.2014.02.015 24642050

[45] Robert-Lachaine X, Dessery Y, Belzile É L, Turmel S, Corbeil P Three-month efficacy of three knee braces in the treatment of medial knee osteoarthritis in a randomized crossover trial. J Orthop Res 2020;38:2262–2271. doi: 10.1002/jor.24634 32077519

[46] Pizzolato C, Reggiani M, Saxby DJ, Ceseracciu E, Modenese L, Lloyd DG Biofeedback for gait retraining based on real-time estimation of tibiofemoral joint Contact Forces. IEEE Trans Neural Syst Rehabil Eng 2017;25:1612–1621. doi: 10.1109/TNSRE.2017.2683488 28436878 PMC5757380

[47] Brouwer RW, van Raaij TM, Verhaar JA, Coene LN, Bierma-Zeinstra SM Brace treatment for osteoarthritis of the knee: a prospective randomized multi-centre trial. Osteoarthritis Cartilage 2006;14:777–783. doi: 10.1016/j.joca.2006.02.004 16563810

[48] Gerus P, Sartori M, Besier TF, Fregly BJ, Delp SL, Banks SA, et al. Subject-specific knee joint geometry improves predictions of medial tibiofemoral contact forces. J Biomech 2013;46:2778–2786. doi: 10.1016/j.jbiomech.2013.09.005 24074941 PMC3888900

[49] Felson DT, Naimark A, Anderson J, Kazis L, Castelli W, Meenan RF The prevalence of knee osteoarthritis in the elderly. The Framingham Osteoarthritis Study. Arthritis Rheum 1987;30:914–918. doi: 10.1002/art.1780300811 3632732

