# Supplementary figures and images for: Effect of a valgus brace on medial tibiofemoral joint contact force in knee osteoarthritis with varus malalignment: A within-participant cross-over randomised study with an uncontrolled observational longitudinal follow-up
Source: PLoS One. 2022 Jun 3;17(6):e0257171. doi: 10.1371/journal.pone.0257171 (PMC9165832; doi:10.1371/journal.pone.0257171)

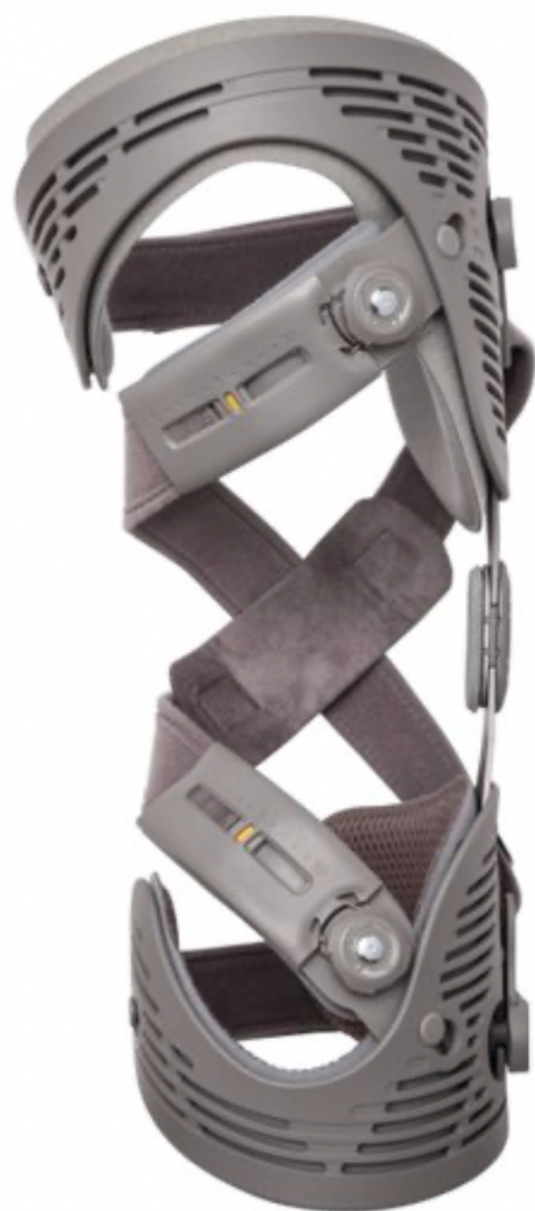

Supplement: S2 Fig — (PDF) [file pone.0257171.s002.pdf]

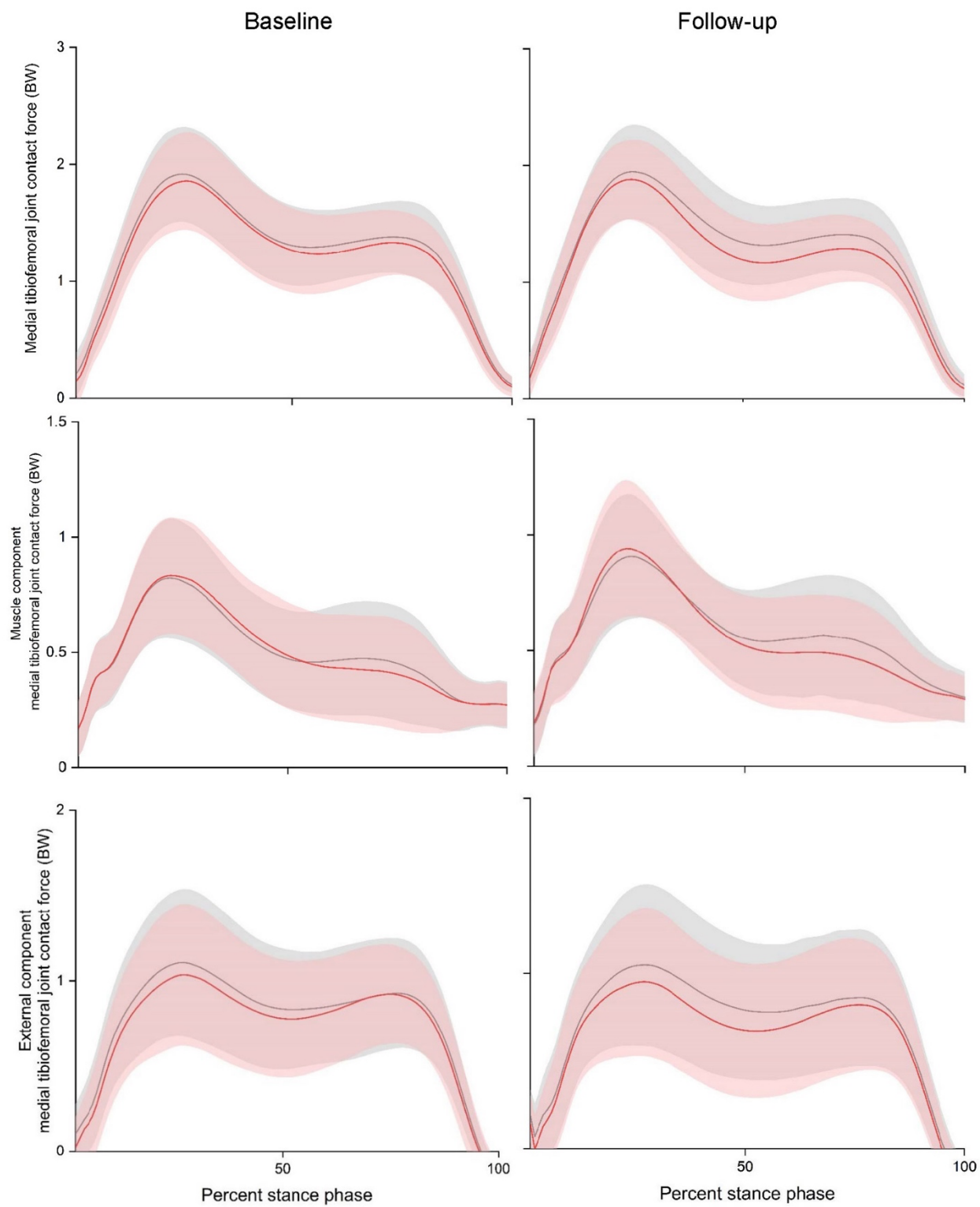

Supplement: S3 Fig — Ensemble average (± standard deviation) of the peak medial tibiofemoral joint contact force (MTCF) and MTCF impulse with the valgus knee brace (red line) and without the valgus knee brace (grey line), along with external and muscle components over a gait cycle at baseline and 8-weeks follow-up. (PDF) [file pone.0257171.s003.pdf]

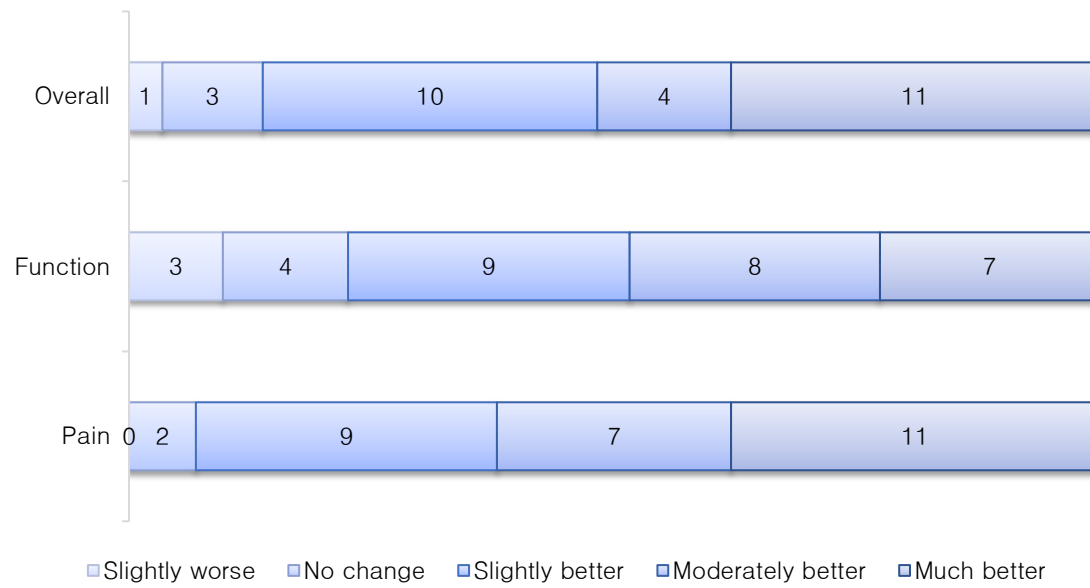

Supplement: S4 Fig — The numbers within the horizontal bars represent the number of participants selecting each option (i.e. slight worse, no change, slightly better, moderately better and much better). One participant who underwent unplanned total knee replacement did not complete these items. (PDF) [file pone.0257171.s004.pdf]

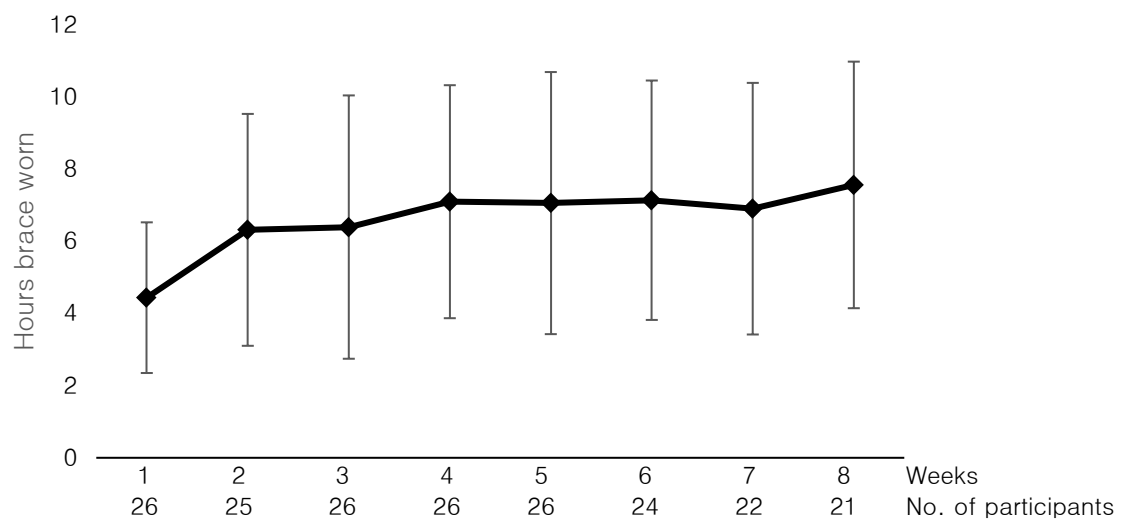

Supplement: S5 Fig — Weekly report of the number of hours the valgus knee brace was worn per week. The number of participants providing weekly data are described, some participants did not provide hours each week for various reasons. (PDF) [file pone.0257171.s005.pdf]

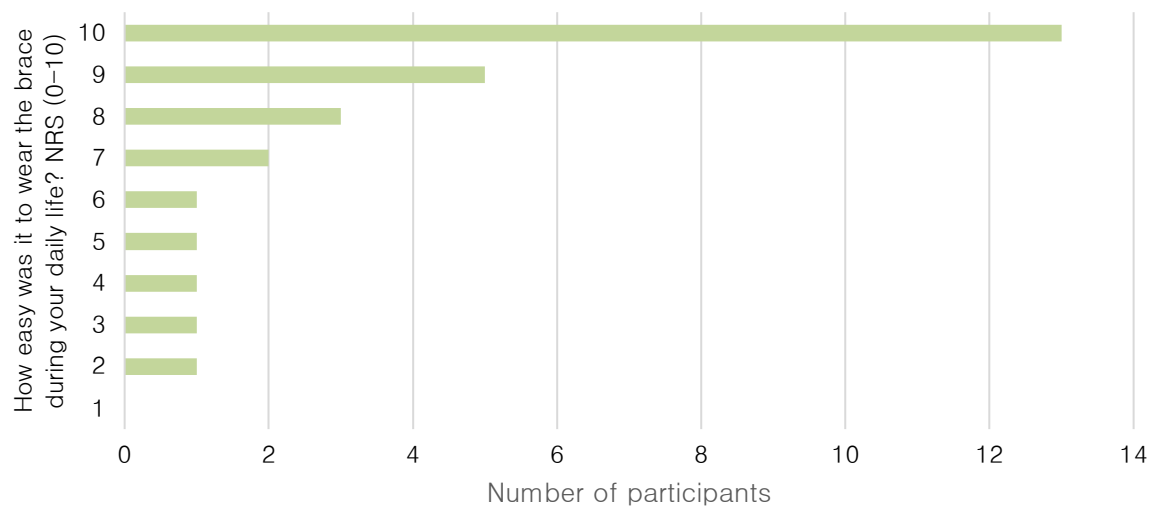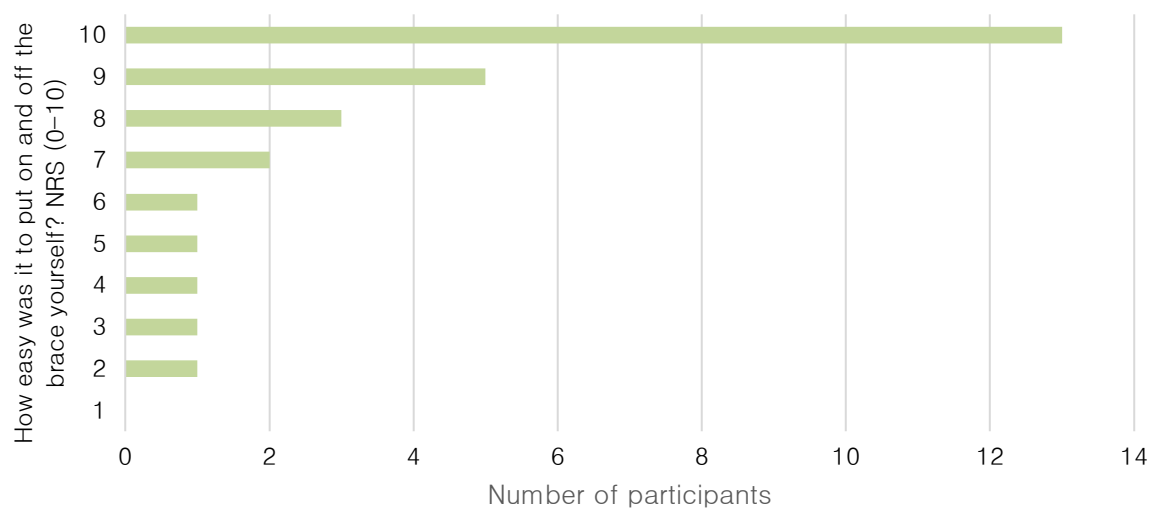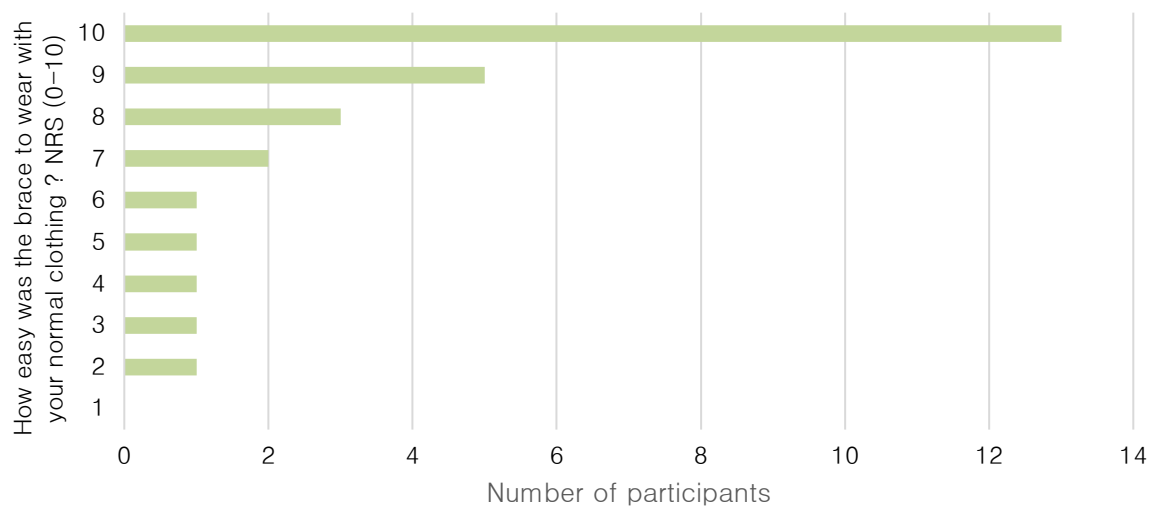

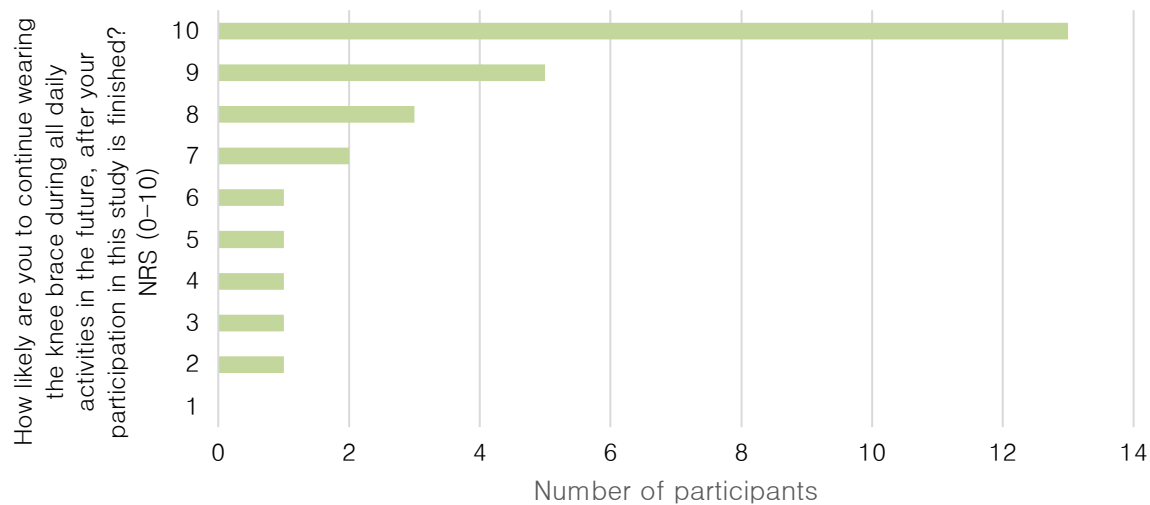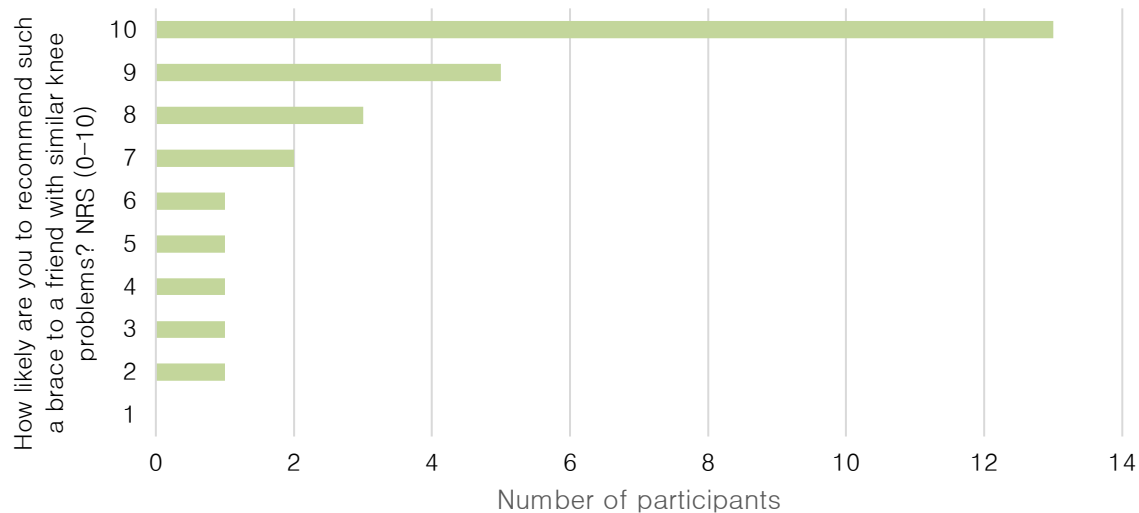

Supplement: S6 Fig — Higher NRS scores indicating more acceptability (10 = extremely easy/likely) and lower scores indicating less acceptabiltiy (0 = not at all easy/likely). Twenty-eight participants responded to these questions at 8-week follow-up. One participant who underwent unplanned total knee replacement did not complete these questions and another participants did not complete these items on the questionairre. (PDF) [file pone.0257171.s006.pdf]
