# Supplementary material for: Effect of a valgus brace on medial tibiofemoral joint contact force in knee osteoarthritis with varus malalignment: A within-participant cross-over randomised study with an uncontrolled observational longitudinal follow-up
Source: PLoS One. 2022 Jun 3;17(6):e0257171. doi: 10.1371/journal.pone.0257171 (PMC9165832; doi:10.1371/journal.pone.0257171)
Supplement: S1 File — (PDF) [file pone.0257171.s008.pdf]

THE UNIVERSITY OF MELBOURNE
HUMAN RESEARCH ETHICS COMMITTEE

APPLICATION SUMMARY FOR APPROVAL OF HUMAN RESEARCH

1. ADMINISTRATION DETAILS

ETHICS ID: **1853473.2**

TITLE: Effects of knee bracing and footwear type on articular contact forces in people with knee osteoarthritis and varus malalignment

**APPLICATION TYPE:**	**Project Application**	**RESPONSIBLE RESEARCHER:**	Hall, DR Michelle Michelle
**RESPONSIBLE HEAG:**	School of Health Sciences	**HESC:**	Psychology Health and Applied Sciences
**ADMINISTERING DEPARTMENT:**	5130 - Physiotherapy	**ADMINISTERING CENTRE:** (if applicable)	Centre for Health, Exercise and Sports Medicine

2. REQUEST FOR AMENDMENT DETAILS

DATE OF SUBMISSION: 22-Jul-2019

1.1 Nature of and Reason for Amendment
It is proposed that an addition be made to the follow-up questionnaire booklet (to include "Part 10 - Brace Feasibility" page 77/78 - highlighted in yellow). This includes 7 questions on a Likert-type scale regarding the practicality of the wearing the brace as instructed during the study and exploring opinions in completing an exercise intervention whilst wearing the knee brace. These questions are aimed at exploring the viability of future research in this area and to evaluate the real-world practicality of the current research project with regards to the knee brace intervention.

1.2 Impact on Documentation
Two additional pages (totalling 7 questions) are requested to be added to the follow-up questionnaire booklet on page 77/78 of the application form. No other changes are intended.

1.3 Possible Inconvenience or Risks to Participants
The follow-up questionnaire (completed during the 8 week laboratory review) is expected to take an additional two minutes to complete as a result of these additional questions. These questions are very short and are in alignment with the style of questions asked in the rest of the questionnaire booklet.

1.4 Actions to be taken by Researchers to Reduce Risks
During emails and/or phone-calls for the research assistant prior to the appointment, participants will be advised of the total time required to complete the follow-up questionnaire booklet, allowing for the increase in time to complete these questions.

1.5 Expected Date of Implementation of Amendment to Research
15-Aug-2019

1.6 Possible Affect on Funding Arrangements
No changes will occur

1.7 Possible Implications for Compliance with Legislative Requirements
No changes will occur

3. PROJECT DETAILS

PROJECT TYPE: Supervised Student Research Project - Masters by Research
Staff Research Project

RESEARCH INVOLVES: Use of drugs or therapeutic devices

Locations other than/in addition to Uni of Melbourne
Administration of ionising radiation
Funded Research (internal or external funding)

BRIEF DESCRIPTION: The primary aims of this study are to use patient-specific, electromyogram-informed neuromusculoskeletal modelling to determine the immediate effects of 1) valgus knee bracing; 2) flat flexible shoes compared to stable supportive shoes and; 3) combination of brace plus flat flexible shoes on medial knee joint force during walking in people with established medial knee osteoarthritis and who have varus malalignment. A secondary aim is to determine the short-term (8 week) effects of the valgus brace on symptoms and knee joint forces.

PROPOSED DURATION OF WHOLE RESEARCH PROJECT: **From:** JAN-2019 **To:** DEC-2020

PROPOSED DATE TO COMMENCE DATA COLLECTION: 01-Mar-2019

4. PERSON DETAILS

Responsible Researcher

**Name**	Hall, Dr Michelle	**Department**	5130 - Physiotherapy
**Person Type**	Staff	**Centre**	Centre for Health, Exercise and Sports Medicine
**Phone Number**	83440556	**Email Address**	halm@unimelb.edu.au

**Qualifications**	Bachelors Degree (Honours), Dublin City University<br>Graduate Certificate, Harvard University<br>PhD, University of Melbourne<br>Masters (Coursework & Research), Iowa State University
**Experience & Skills Relevant to the Project**	Dr Michelle Hall is a post-doctoral research fellow at the Centre for Health, Exercise and Sports Medicine at The University of Melbourne. Dr Hall has extensive experience in Biomechanics research, running several randomised control trials investigating the effects of physical training on joint health in clinical populations. She is also the lead contact with our industry partners Ossur.<br>Dr Hall is the responsible researcher for this project and will be responsible for ensuring that this project is conducted in accordance with University's human research ethics guidelines, the national Statement on Ethical Conduct in Human Research, and that the project adheres to the University's HREC protocols. Dr Hall will also be providing supervision to the student researcher on this project.
**Additional Training Required**	
**Ethics Training Already Undertaken**	Research Integrity Online Training (RIOT)

Student Researcher

**Name**	Starkey, Scott	**Department**	5130 - Physiotherapy
**Person Type**	Student	**Centre**	Centre for Health, Exercise and Sports Medicine
**Phone Number**		**Email Address**	s.starkey@student.unimelb.edu.au/Alt:scott.starkey@unimelb.edu.au

**Qualifications**	Bachelor of Biomedicine, University of Melbourne (2014)<br>Doctor of Physiotherapy, University of Melbourne (2017)
**Experience & Skills Relevant to the Project**	Scott has experience in performing neuromusculoskeletal modelling techniques to estimate knee joint contact forces. He also has qualifications in physiotherapy and is experienced in the clinical testing and management of people with knee osteoarthritis
**Additional Training Required**	
**Ethics Training Already Undertaken**	Research Integrity Online Training (RIOT), University of Melbourne (2018)
**Student Supervisor(if applicable)**	Michelle Hall, Rana Hinman, Tim Wrigley, David Saxby

Co researcher

**Name**	Paterson, Dr Kade	**Department**	5130 - Physiotherapy
**Person Type**	Staff	**Centre**	Centre for Health, Exercise and Sports Medicine

**Phone Number**	8344 0425	**Email Address**	kade.paterson@unimelb.edu.au
**Qualifications**	Bachelors Degree, La Trobe University<br>Bachelors Degree (Honours), Australian Catholic University<br>PhD, Australian Catholic University		
**Experience & Skills Relevant to the Project**	Dr Paterson is an experienced podiatrist who maintains an active clinical practice and brings specialised biomechanical expertise in foot function and foot interventions. He will oversee the footwear intervention component according to pre-specified criteria and manage any footwear-related adverse events.		
**Additional Training Required**			
**Ethics Training Already Undertaken**	Research Integrity Online Training (RIOT)		

Co researcher

**Name**	Hinman, Prof Rana	**Department**	5130 - Physiotherapy
**Person Type**	Staff	**Centre**	
**Phone Number**	83443223	**Email Address**	ranash@unimelb.edu.au
**Qualifications**	Bachelors Degree (Honours), University of Melbourne<br>PhD, University of Melbourne		
**Experience & Skills Relevant to the Project**	Prof Hinman is a research physiotherapist who has conducted 20 RCTs in musculoskeletal conditions, most in knee OA. She will provide supervision to the research student involved in this project, as well as grade the x-rays obtained during this study.		
**Additional Training Required**			
**Ethics Training Already Undertaken**	Research Integrity Online Training (RIOT)		

Co researcher

**Name**	Saxby, DR David	**Department**	Griffith University
**Person Type**	External	**Centre**	
**Phone Number**		**Email Address**	d.saxby@griffith.edu.au
**Qualifications**	PhD (2016), Centre for Musculoskeletal Research at the School of Allied Health Sciences, Griffith University, Australia.<br>MSc (2010), School of Human Kinetics, University of Ottawa, Canada.<br>BSc Hon (2008), School of Human Kinetics, University of Ottawa, Canada.		
**Experience & Skills Relevant to the Project**	Dr David John Saxby is a faculty member of the School of Allied Health Science at Griffith University. Both his MSc and PhD were in Biomechanics, and he has >500 hours of in-laboratory training and experience in Biomechanics research. David has extensive experience in neuromusculoskeletal modelling and magnetic resonance imaging segmentation techniques. He will also be providing supervision for the research student involved in this project.		
**Additional Training Required**			
**Ethics Training Already Undertaken**			

5. ADDITIONAL QUESTIONS

5.1 Sponsored Projects

Source of Funding: External

External Sponsors Identified: Yes

Sponsors: Commonwealth Innovation Connection Grant
Ossür (Industry Partner)

Participants Informed of Funding Source: Yes - Participants will be informed about sources of funding in writing within the distributed plain language statement.

5.2 Clinical Trials

Registered in public trial registry: No

Registry Name:

Registry Number:

Date of Registration:

Project Name recorded in registry:

Name of researcher in whose name the project is registered:

5.3 Drug or Therapeutic Device Trials

Type of Trial: Routine

Phase (if applicable): N/A

5.4 Location of Research

Location Where Research Will Be Carried Out: External sites within Australia
University of Melbourne

Category of External Location: Hospitals

5.5 Other Approvals Required (other than ethics clearances)

Approvals Required: Required

Approvals Source Identified: Yes

Approval Required From:

Approval Required From	Approval Status	Date Approval Granted	Special Conditions
Murdoch Children's Research Institute, The Royal Children's Hospital	Approved		
**Comments**			

Comments: The Murdoch Children's Research Institute has confirmed the ability to provide Magnetic Resonance Imaging services for this study, however the acquisition of services agreement is awaiting review and appropriate signatures at their business development and legal office. A letter from the Group Leader at Developmental Imaging, Murdoch Children's Research Institute is provided in the attachments outlining this process.

5.6 Other Ethic Clearances/Details of Multicentre Research

Other Clearances Required: Not required

Responsible HREC:

Comments:

6. ATTACHMENTS

PLEASE ENSURE YOU ATTACH A PAPER COPY OF EACH OF THE FOLLOWING ATTACHMENTS:

Category	Description	Attached Via Themis	Hard Copy Only
Additional Module	For knee brace	Yes	No
Additional Module		Yes	No
Advertisement	Examples of advertising for study recruitment	Yes	No
Application	Main ethics application form for the proposed study	Yes	No
Interview		Yes	No
Miscellaneous	1853473.1_Approval letter.pdf	Yes	No
Miscellaneous	1853473.1_Response to HESC Review.pdf	Yes	No
Miscellaneous	In progress agreement and letter from Developmental imaging, Murchoch's Children's Research Institute confirming ability to provide services	Yes	No
Miscellaneous		Yes	No
Plain Language Statement	Information letter, plain language statement, consent form and additional information regarding magnetic resonance imaging	Yes	No
Questionnaire/Survey	All questionnaires to be taken at baseline and at 8 week follow-up	Yes	No
Questionnaire/Survey	Log book for participants to complete during 8 weeks of brace intervention	Yes	No
Questionnaire/Survey	Online form for initial screening of participants recruited from the community	Yes	No

7. SIGNATURE

RESPONSIBLE RESEARCHER

Name	Signature	Date
Dr Michelle Hall		

HEAG/DEPARTMENT USE ONLY

Please tick ONE of the following:

The HEAG/Head of Department recommends the amendment for approval by the HESC.	
The HEAG approves this amendment as the project was previously approved as a minimal risk project or as a project within a program and the amendment presents no additional risks.	

Comments / provisos:

HEAG CHAIR

Name of HEAG Chair	Signature	Date
Dr Snezana Kusljic		

[Note: If the HEAG Chair is also a researcher involved in this project, the Request for Amendment should be signed by another authorised member of the HEAG. If the Department does not have access to a HEAG the Request for Amendment should be signed by the Head of Department]

THE UNIVERSITY OF MELBOURNE
HUMAN RESEARCH ETHICS COMMITTEE

APPLICATION SUMMARY FOR APPROVAL OF HUMAN RESEARCH

1. ADMINISTRATION DETAILS

ETHICS ID: **1853473.1**

TITLE: Effects of knee bracing and footwear type on articular contact forces in people with knee osteoarthritis and varus malalignment

**APPLICATION TYPE:**	**Project Application**	**RESPONSIBLE RESEARCHER:**	Hall, DR Michelle Michelle
**RESPONSIBLE HEAG:**	School of Health Sciences	**HESC:**	Psychology Health and Applied Sciences
**ADMINISTERING DEPARTMENT:**	5130 - Physiotherapy	**ADMINISTERING CENTRE:** (if applicable)	Centre for Health, Exercise and Sports Medicine

2. PROJECT DETAILS

PROJECT TYPE: Supervised Student Research Project - Masters by Research
Staff Research Project

RESEARCH INVOLVES: Use of drugs or therapeutic devices
Locations other than/in addition to Uni of Melbourne
Funded Research (internal or external funding)
Administration of ionising radiation

BRIEF DESCRIPTION: The primary aims of this study are to use patient-specific, electromyogram-informed neuromusculoskeletal modelling to determine the immediate effects of 1) valgus knee bracing; 2) flat flexible shoes compared to stable supportive shoes and; 3) combination of brace plus flat flexible shoes on medial knee joint force during walking in people with established medial knee osteoarthritis and who have varus malalignment. A secondary aim is to determine the short-term (8 week) effects of the valgus brace on symptoms and knee joint forces.

PROPOSED DURATION OF WHOLE RESEARCH PROJECT: **From:** JAN-2019 **To:** DEC-2020

PROPOSED DATE TO COMMENCE DATA COLLECTION: 01-Mar-2019

3. PERSON DETAILS

Responsible Researcher

**Name**	Hall, Dr Michelle	**Department**	5130 - Physiotherapy
**Person Type**	Staff	**Centre**	Centre for Health, Exercise and Sports Medicine
**Phone Number**	83440556	**Email Address**	halm@unimelb.edu.au
**Qualifications**	Bachelors Degree (Honours), Dublin City University<br>Graduate Certificate, Harvard University<br>PhD, University of Melbourne<br>Masters (Coursework & Research), Iowa State University		
**Experience & Skills Relevant to the Project**	Dr Michelle Hall is a post-doctoral research fellow at the Centre for Health, Exercise and Sports Medicine at The University of Melbourne. Dr Hall has extensive experience in Biomechanics research, running several randomised control trials investigating the effects of physical training on joint health in clinical		

	populations. She is also the lead contact with our industry partners Ossur.<br>Dr Hall is the responsible researcher for this project and will be responsible for ensuring that this project is conducted in accordance with University's human research ethics guidelines, the national Statement on Ethical Conduct in Human Research, and that the project adheres to the University's HREC protocols. Dr Hall will also be providing supervision to the student researcher on this project.
**Additional Training Required**	
**Ethics Training Already Undertaken**	Research Integrity Online Training (RIOT)

Student Researcher

**Name**	Starkey, Scott	**Department**	5130 - Physiotherapy
**Person Type**	Student	**Centre**	Centre for Health, Exercise and Sports Medicine
**Phone Number**		**Email Address**	s.starkey@student.unimelb.edu.au/Alt:scott.starkey@unimelb.edu.au

**Qualifications**	Bachelor of Biomedicine, University of Melbourne (2014)<br>Doctor of Physiotherapy, University of Melbourne (2017)
**Experience & Skills Relevant to the Project**	Scott has experience in performing neuromusculoskeletal modelling techniques to estimate knee joint contact forces. He also has qualifications in physiotherapy and is experienced in the clinical testing and management of people with knee osteoarthritis
**Additional Training Required**	
**Ethics Training Already Undertaken**	Research Integrity Online Training (RIOT), University of Melbourne (2018)
**Student Supervisor(if applicable)**	Michelle Hall, Rana Hinman, Tim Wrigley, David Saxby

Co researcher

**Name**	Paterson, Dr Kade	**Department**	5130 - Physiotherapy
**Person Type**	Staff	**Centre**	Centre for Health, Exercise and Sports Medicine
**Phone Number**	8344 0425	**Email Address**	kade.paterson@unimelb.edu.au

**Qualifications**	Bachelors Degree, La Trobe University<br>Bachelors Degree (Honours), Australian Catholic University<br>PhD, Australian Catholic University
**Experience & Skills Relevant to the Project**	Dr Paterson is an experienced podiatrist who maintains an active clinical practice and brings specialised biomechanical expertise in foot function and foot interventions. He will oversee the footwear intervention component according to pre-specified criteria and manage any footwear-related adverse events.
**Additional Training Required**	
**Ethics Training Already Undertaken**	Research Integrity Online Training (RIOT)

Co researcher

**Name**	Hinman, Prof Rana	**Department**	5130 - Physiotherapy
**Person Type**	Staff	**Centre**	
**Phone Number**	83443223	**Email Address**	ranash@unimelb.edu.au

**Qualifications**	Bachelors Degree (Honours), University of Melbourne<br>PhD, University of Melbourne
**Experience & Skills Relevant to the Project**	Prof Hinman is a research physiotherapist who has conducted 20 RCTs in musculoskeletal conditions, most in knee OA. She will provide supervision to the research student involved in this project, as well as grade the x-rays obtained during this study.
**Additional Training Required**	
**Ethics Training Already Undertaken**	Research Integrity Online Training (RIOT)

Co researcher

**Name**	Saxby, DR David	**Department**	Griffith University
**Person Type**	External	**Centre**	

Phone Number		Email Address	d.saxby@griffith.edu.au
**Qualifications**	PhD (2016), Centre for Musculoskeletal Research at the School of Allied Health Sciences, Griffith University, Australia.<br>MSc (2010), School of Human Kinetics, University of Ottawa, Canada.<br>BSc Hon (2008), School of Human Kinetics, University of Ottawa, Canada.		
**Experience & Skills Relevant to the Project**	Dr David John Saxby is a faculty member of the School of Allied Health Science at Griffith University. Both his MSc and PhD were in Biomechanics, and he has >500 hours of in-laboratory training and experience in Biomechanics research. David has extensive experience in neuromusculoskeletal modelling and magnetic resonance imaging segmentation techniques. He will also be providing supervision for the research student involved in this project.		
**Additional Training Required**			
**Ethics Training Already Undertaken**			

4. ADDITIONAL QUESTIONS

4.1 Sponsored Projects

Source of Funding: External

External Sponsors Identified: Yes

Sponsors: Commonwealth Innovation Connection Grant
Ossür (Industry Partner)

Participants Informed of Funding Source: Yes - Participants will be informed about sources of funding in writing within the distributed plain language statement.

4.2 Clinical Trials

Registered in public trial registry: No

Registry Name:

Registry Number:

Date of Registration:

Project Name recorded in registry:

Name of researcher in whose name the project is registered:

4.3 Drug or Therapeutic Device Trials

Type of Trial: Routine

Phase (if applicable): N/A

4.4 Location of Research

Location Where Research Will Be Carried Out: External sites within Australia
University of Melbourne

Category of External Location: Hospitals

4.5 Other Approvals Required (other than ethics clearances)

Approvals Required: Required

Approvals Source Identified: Yes

Approval Required From:

Approval Required From	Approval Status	Date Approval Granted	Special Conditions
Murdoch Children's Research Institute, The Royal Children's Hospital	Under Review		
**Comments**			

Comments: The Murdoch Children's Research Institute has confirmed the ability to provide Magnetic Resonance Imaging services for this study, however the acquisition of services agreement is awaiting review and appropriate signatures at their business development and legal office. A letter from the Group Leader at Developmental Imaging, Murdoch Children's Research Institute is provided in the attachments outlining this process.

4.6 Other Ethic Clearances/Details of Multicentre Research

Other Clearances Required: Not required

Responsible HREC:

Comments:

5. ATTACHMENTS

PLEASE ENSURE YOU ATTACH A PAPER COPY OF EACH OF THE FOLLOWING ATTACHMENTS:

Category	Description	Attached Via Themis	Hard Copy Only
Additional Module	For knee brace	Yes	No
Additional Module		Yes	No
Advertisement	Examples of advertising for study recruitment	Yes	No
Application	Main ethics application form for the proposed study	Yes	No
Interview		Yes	No
Miscellaneous	In progress agreement and letter from Developmental imaging, Murchoch's Children's Research Institute confirming ability to provide services	Yes	No
Plain Language Statement	Information letter, plain language statement, consent form and additional information regarding magnetic resonance imaging	Yes	No
Questionnaire/Survey	All questionnaires to be taken at baseline and at 8 week follow-up	Yes	No
Questionnaire/Survey	Log book for participants to complete during 8 weeks of brace intervention	Yes	No
Questionnaire/Survey	Online form for initial screening of participants recruited from the community	Yes	No

HUMAN RESEARCH ETHICS

PROJECT APPLICATION FORM

How To Use This Form

1. **Consider and refer to relevant guidelines and regulations.**
References to specific guidelines are provided, with hyperlinks, throughout this form. The primary guide for human research ethics in Australia is the ***National Statement on Ethical Conduct in Human Research (2007) - Updated 2018***. Human research ethics applications at the University of Melbourne are reviewed and approved under the warrant of the *National Statement*. References to the *National Statement* are abbreviated (e.g. ***NS* §2.1**.)

2. **Use plain English.**
Use clear, non-technical language in your application. Be concise. Spell out the first instances of acronyms and abbreviations. Avoid jargon. Do not repeat information. Following these directions ensures effective review of your application. It will avoid unnecessary delays which result if applications are not clear and concise.

3. **Consider ethical principles.**
Your application will be reviewed according to the principles of ethical research outlined in the *National Statement*, namely:
   - **Research Merit and Integrity** (***NS* §1.1 - §1.3**)
   - **Justice** (***NS* §1.4 - §1.5**)
   - **Beneficence** (***NS* §1.6 - §1.9**)
   - **Respect** (***NS* §1.10 - §1.13**)

4. **Use the current version of the application form.**
Ensure that you are using the current version by downloading **this form** each time you prepare a new application.

5. **Detailed instructions for specific questions are available online.**
If you are unsure about how best to answer a particular question, consult the Human Research Ethics **Guidance Document**. That document provides detailed guidance on how to answer specific questions in this form.

6. **Where possible, avoid printing this form.**
Consult your HEAG to find out if they still require hard copies of your application. If you must print this form, consider printing double-sided and in grayscale (black and white).

7. **Save your completed application as a PDF and upload it to Themis.**
Refer to your local Human Ethics Advisory Group (**HEAG**) for detailed instructions on how and when to submit your application.

ANSWER ALL OF THE QUESTIONS IN THIS FORM

**Ethics ID number:** *(assigned by Themis)*	1853473.1	
**Project Title:** *(as recorded in Themis)*	Effects of knee bracing and footwear type on articular contact forces in people with knee osteoarthritis and varus malalignment	
**Responsible Researcher:** *(as recorded in Themis)*	Dr Michelle Hall	
**Application Type:** *(mark with an "X")*		**Minimal Risk**
	X	**Standard Project**

1. Project Details

1.1 Project Summary	Summarise your research project in plain language. **[Limit: 300 words]**

A) Aims and Objectives

[Briefly describe the broad aims and objectives of this project.]

The primary aims of this study are to use patient-specific, electromyogram-informed neuromusculoskeletal modelling to determine the immediate effects of 1) valgus knee bracing; 2) flat flexible shoes compared to stable supportive shoes and; 3) combination of brace plus flat flexible shoes on medial knee joint contact force during walking in people with established medial knee osteoarthritis and who have varus malalignment. A secondary aim is to determine the short-term (8 week) effects of the valgus brace on symptoms and joint forces.

B) Key Question(s)

[What question, or questions, does the project intend to examine? Where relevant, state the specific hypothesis to be tested.]

This study will test the hypotheses that medial knee joint force during walking will reduce 1) immediately wearing flat flexible walking shoes compared to stable supportive walking shoes; 2) immediately wearing a valgus knee brace compared to not wearing a valgus knee brace; 3) more with the combination of brace/flat flexible shoes than either intervention alone and 4) braced walking following daily use of a valgus knee brace over 8 weeks, compared to braced walking at baseline.

C) Research Design

[Outline the research design/approach. In particular,note the type(s) of participants, and type(s) of data collection.]

To test the immediate effects of the knee brace and two types of footwear, a within-participant study design will be used. To test the effect of daily valgus knee brace use over 8 weeks an observational within-subjects study design will be used. Thirty participants over the age of 50 years with clinically diagnosed medial tibiofemoral osteoarthritis and varus malalignment will be recruited from an existing database and if required, the community.

Potentially eligible participants who consent will undergo a baseline x-ray (if required, we expect that some participants will have had a knee x-ray in previous 12 months) to determine the location and severity of osteoarthritis, and knee alignment. Only eligible participants (knee pain > 3 months on most days; minimum pain score 4 on a 11-point numerical rating score; radiographical medial osteoarthritis and varus knee alignment) will undergo a magnetic resonance (MR) image of the affected knee. These images will be used to create a three-dimensional (3D) anatomical knee surface to use in estimates of joint contact forces. Participants will be required to attend the human movement laboratory at the Centre for Health, Exercise and Sports Medicine for assessment. Data collection will include 3D gait/functional analysis, consisting of marker trajectories, ground reaction force (GRF) plate data, and muscle activation patterns via electromyograms (EMG). Maximal muscle contractions of the major lower limb muscles will also be obtained.[1] Other data collected will include self-reported symptoms, quality of life, self- efficacy any co-interventions used during the 8-week study period, as well as adherence to wearing the brace and any adverse effects.

Specific Guidelines Checklist		
**Type an "X" in the left-hand column beside all items that apply to your research project.** Linked sections of the National Statement contain relevant guidelines and requirements that you need to address when completing your application.		
	**Children and/or young people (< 18 years old)** will be recruited as participants.	→ **Refer to *NS §4.2*.**
	**People in dependent or unequal relationships** will be recruited as participants.<br>(There are pre-existing relationships between participants and researchers, or between participants and others involved in facilitating or implementing the research. E.g. student/teacher, patient/doctor, employee/employer.)	→ **Refer to *NS §4.3*.**
	**People in countries other than Australia** will be recruited as participants.	→ **Refer to *NS §4.8*.**
	One or more of the following describes the research project:<br>• it will be about **Aboriginal and/or Torres Strait Islander individuals or peoples**, their health, or their culture(s), language(s) or histories;<br>• it will be about the impact(s) or effect(s) of some phenomenon or phenomena on **Aboriginal and/or Torres Strait Islander individuals or peoples;**<br>• it will *specifically target* **Aboriginal and/or Torres Strait Islander people** to be recruited as participants;<br>• it will be conducted in a geographic location where a significant number of the population are likely to be **Aboriginal and/or Torres Strait Islander.**	→ **Refer to *NS §4.7*.**<br>→ **Refer to *Ethical Conduct in Research with Aboriginal and Torres Strait Islander Peoples and Communities: Guidelines for Researchers and Stakeholders (2018)*.**<br>→ **Refer to Guidelines for Ethical Research in Australian Indigenous Studies**<br>→ **This application is ineligible for minimal risk review.**
	One or both of the following describes the research project:<br>• it will *specifically target* **women who are pregnant** to be recruited as participants;<br>• it will be focused on **women who are pregnant and/or the human foetus** (including human foetal tissue or human embryos).	→ **Refer to *NS §4.1*.**<br>→ **This application is ineligible for minimal risk review.**
	**People who may be involved in illegal activities** will be recruited as participants, ***and the research project could potentially expose such activities.***	→ **Refer to *NS §4.6*.**<br>→ **This application is *likely* ineligible for minimal risk review.**
	**People with cognitive impairment, intellectual disability, or mental illness** will be recruited as participants.	→ **Refer to *NS §4.5*.**<br>→ **This application is ineligible for minimal risk review.**
	**People who are highly dependent on medical care** will be recruited as participants.	→ **Refer to *NS §4.4*.**<br>→ **This application is ineligible for minimal risk review.**
**X**	**None of the above** applies to this research project.	

Additional Modules Checklist		
**Type an "X" in the left-hand column beside all items that apply to your research project.** This checklist will help you determine if you need to complete any other modules in addition to this application form. Linked sections of the *National Statement* contain relevant guidelines and requirements that you need to address when completing this form and any applicable additional modules.		
	This research project will involve the **creation of a databank** (i.e. your stored data will be made available to other parties for secondary use in future research projects).	→ **Refer to NS §3.1.**<br>→ **Complete and attach the *Privacy and Databanks Module*.**
	This research project will involve the **collection of information for a databank** (i.e. your stored data will be made available to other parties for secondary use in future research projects).	→ **Refer to NS §3.1.**<br>→ **Complete and attach the *Privacy and Databanks Module*.**
	This research project will involve **accessing information from an existing databank** (i.e. you will be accessing and making use of stored data that was previously collected – not for this specific project – by other parties).	→ **Refer to NS §3.1.**<br>→ **Complete and attach the *Privacy and Databanks Module*.**
	This research project will involve obtaining identifiable (or potentially identifiable) **personal information (including health information)** about individuals ***without their consent.***	→ **Complete and attach the *Privacy and Databanks Module*.**
	This research project will involve the collection and/or use of **human tissue/biological samples or materials** (e.g. blood, saliva, cheek swabs, hair, human embryonic or foetal tissue).	→**Refer to *NS §3.2*.**<br>→ **Complete and attach the *Body Tissue and Genetic Research Module*.**
	This research project will involve **genomic research.**	→ **Refer to *NS §3.3*.**<br>→ **This application is ineligible for minimal risk review.**<br>→ **Complete and attach the *Body Tissue and Genetic Research Module*.**
X	This research project will involve **medical interventions, therapies or trials.**	→ **Refer to NS §3.1.**<br>→ **This application is ineligible for minimal risk review.**<br>→ **Complete and attach the *Interventions, Therapies and Trials Module*.**
X	This research project will involve **administration of ionising radiation.**	→ **Complete and attach the *Ionising Radiation Module*.**
	**None of the above** applies to this research project.	

2. Background and Method

2.1 Background and Significance	Provide a summary of background information. Explain the significance of the proposed research in the context of this background. **Refer to *NS §5.2.5* and NS §3.1**<br>**[Limit: 500 words]**

A) Background:

[What is the current state of research/knowledge/discourse in this area?]

Knee osteoarthritis is a global public health problem, causing pain and disability to those affected.[2] This disability is even more pronounced in a sub-group of patients with medial knee osteoarthritis and varus malalignment.[3] Health care costs are substantial and are forecasted to reach $2.9 billion annually in Australia by 2030.[2] As there is no cure, there is a need for effective interventions to prevent progression of this disease.

Importantly, higher medial knee joint loading during walking is associated with structural disease progression in people with medial knee osteoarthritis.[4] Interventions aimed at reducing medial knee loading are therefore an important research area. External aids such as knee braces provide a non-invasive strategy to directly modulate these loads. Valgus knee braces such as the Ossur Unloader One© are designed to apply an external valgus force to the knee during walking and other tasks, potentially altering knee joint loading. Previous research suggests that valgus knee braces can significantly improve quality of life and can potentially delay the need for surgery.[5] Footwear type is another intervention that can alter knee joint loads. Footwear forms an integral component of daily life and is considered a research priority for osteoarthritis.[7] Limited evidence suggests that flat flexible shoes lower knee joint loads more than stable supportive shoe styles, even though stable supportive shoe styles are typically worn by people with knee osteoarthritis.[6] Furthermore, clinical guidelines recommend that clinicians provide advice regarding appropriate footwear, yet do not specify the footwear type.[7]

Critically, previous research has used the external knee adduction moment as an estimate of knee loading. However, an important limitation of using external moments to infer internal joint loading is that external moments do not account for the contribution of muscle activation to internal joint loading. In fact, muscle forces inferred through muscle activation patterns account for a substantial proportion (>50%) of medial tibiofemoral contact force during walking.[8] Given all current studies in this population share this limitation (i.e. estimate net moments), we cannot conclusively determine whether bracing or certain footwear types are able to reduce articular knee load and potentially osteoarthritis progression.

B) Significance of This Research:

[Explain the significance of the proposed research project in light of existing research, knowledge or understanding. How does your research help to fill a gap in the literature? You may include relevant references, within the word limit.]

We will use a cutting-edge neuromusculoskeletal modelling technique that integrates both internal muscle forces and external forces to provide an estimate of medial tibiofemoral contact force.[8] Furthermore, by using MR imaging data, we are able create 3D anatomical knee surfaces to use in our modelling, which provides more accurate estimates than a generic model. In addition to providing a more valid estimation of knee articular loading than traditional techniques, our modelling provides insight into whether and in what way people modify their articular loading patterns. The outcomes of this study will provide an in depth understanding of how contact forces within our knee joint responds to bracing and footwear types. Findings will inform clinicians whether a knee brace, footwear or combination of both is best at reducing harmful knee loads and will thus assist clinical decision-making and ultimately benefit patients with knee osteoarthritis.

References:

1. Agostini V, et al. (2014). Gait parameters and muscle activation patterns at 3, 6 and 12 months after total hip arthroplasty. *J Arthroplasty,* 29(6), 1265-72.
2. Ackerman IN, et al. (2016). Counting the Cost: The current and future burden of arthritis.
3. Sharma L, et al. (2010). Varus and valgus alignment and incident and progressive knee osteoarthritis. *Ann Rheum Dis*, 69(11), 1940-1945.
4. Bennell KL, et al. (2011). Higher dynamic medial knee load predicts greater cartilage loss over 12 months in medial knee osteoarthritis. *Ann Rheum Dis,* 70(10), 770-4.
5. Mistry DA, et al. (2018). An Update on unloading knee braces in the treatment of unicompartmental knee osteoarthritis from the last 10 years: a literature review. *Surgery Journal,* 4(3), e110-e8.
6. Paterson KL, et al. (2017). Effects of footwear on the knee adduction moment in medial knee osteoarthritis: classification criteria for flat flexible vs stable supportive shoes. *Osteoarthr Cartil,* 25, 234–241.
7. National Clinical Guideline Centre. (2014). Osteoarthritis: Care and management in adults. National Institute for Health and Care Excellence, London.
8. Winby CR, et al. (2009). Muscle and external load contribution to knee joint contact loads during normal gait. *J Biomech,* 42(14),2294-300.

2.2 Research Design and Method	Provide details of your research design and your proposed method. **Refer to *NS §5.2.5 - §5.2.6* and NS §3.1**<br>**Attach a copy of any measures, scales, questionnaires, survey instruments (including online surveys), interview questions/themes, and/or focus group topics/questions to be used.**

A) Participants (or Recruitment Targets, such as medical records):

[Describe the sample, i.e. the intended participants or recruitment targets. Explain the basis on which this sample was chosen. Include the number and age range and any other relevant demographic characteristics of participants, as well as any eligibility constraints (i.e. inclusion/exclusion criteria). If the project involves using records or previously-•collected data/samples, rather than direct contact with human participants, state that.]

Thirty participants with clinically diagnosed medial knee osteoarthritis and varus malalignment will be recruited. We aim to detect an immediate bracing/footwear effect size of 0.3 for medial knee joint contact force. Assuming power of 80% and alpha of 0.05, a correlation between measurement of 0.875 a sample of at least 24 participants is required. Allowing for 20% loss to follow-up, we will recruit 30 participants into the study.

Inclusion criteria

Inclusion criteria are based on the American College of Rheumatology clinical and radiographic criteria for knee osteoarthritis[1]

- Age ≥ 50 years;
- Report knee pain on most days of the past month for >3 months;
- Pain on most days (5-7 days/week or 20-30 days/month) in the last month;
- Report a minimum pain score of 4 on an 11-point numeric rating scale during walking over the previous week;
- Willing to wear a knee brace during daily activities, every day for 8 weeks;
- Wear a Female shoe size 6-11 US or male shoe size 8-13 US;
- Able to fit shoes into normal width (all shoes in this study are of normal width);
- Able to travel to undergo x-ray (if required) / MR examinations, and to attend the Centre for Health, Exercise and Sports Medicine (CHESM) human movement lab at the University of Melbourne for testing;
- Radiographic medial tibiofemoral joint osteoarthritis*;

*Specific inclusion criteria based on a posteroanterior weight-bearing radiograph are i) Kellgren/Lawrence[2] grade ≥ 2; ii) anatomic axis angle of <181 degrees for females or <183 degrees for males, indicating varus alignment based on mechanical axis values using the sex-specific regression equation[3]; iii) medial tibiofemoral joint narrowing grade greater than lateral tibiofemoral joint narrowing grade; iv) medial compartment osteophyte grade greater than or equal to lateral compartment osteophyte grade.

Exclusion criteria:

- Knee or hip replacement on affected side or high tibial osteotomy;
- Any knee surgery including arthroscopes in the past 6 months;
- Have a body mass index ≥ 36 kg/m^2 (due to difficulties in three-dimensional gait analysis);
- Awaiting or planning any back or lower-limb surgery within the next 3 months;
- Plans to see an orthopaedic surgeon about knee within the next 8 weeks;
- Current or past (within 3 months) oral or intra-articular corticosteroid use;
- Systemic arthritic conditions;

- Work restrictions of other commitments that would restrict wearing a knee brace during daily activities;
- Current (or within past 6 months) muscular, joint or neurological condition;
- Current (or within past 6 months), or intention to use with the next 8 weeks, a knee brace, walking stick or gait aid;
- Absolute contraindications to MR imaging (cardiac pacemaker; surgery to head, heart, brain or eyes; any metal fragments in eyes; any operations involving the use of metal or electronic implants, plate, stents or valves; any surgery to any part of the body in the past two months; deep brain stimulator; pregnancy; currently breastfeeding; history of renal disease; diabetes).

B) Recruitment:

[Describe how recruitment will occur. Explain how potential participants will be identified and approached. Who will do this? Refer to NS §2.2. If you will be using records or data only, and you will be completing the Privacy and Databanks Module, state "N/A." If you will be using records or data only, but you will not be completing the Privacy and Databanks Module, explain how the records/data will be identified, collected and accessed.]

All stages of screening and recruitment will be performed by the research team. Participants will be initially recruited from the CHESM volunteer database. This is a database of volunteers who have previously participated in CHESM studies and have agreed to be contacted for future research studies. Although it is expected that most participants will be recruited through the CHESM volunteer database, if required, participants will also be recruited from the community via advertisements, print/radio/social medial and clinicians (attachment 1). Participants recruited from the community will be screened initially by an online form (SurveyGizmo, attachment 2). All participants will then undergo a telephone interview conducted by the student researcher (attachment 3). If potentially eligible following telephone screening, participants will be emailed/mailed an information letter, plain language statement and consent form (attachment 4). A phone call will be made by the student researcher after at least 7 days if no response has been received. If written consent is received, those potentially eligible participants that have not had an x-ray in the past 12 months will then be booked in to receive bilateral x-rays of their knees. Screening of all x-rays will be conducted by the research team with the following criteria: presence of mild to severe medial radiographic knee OA; varus malalignment. If potentially eligible participants pass x-ray screening, they will then be enrolled into the study and booked in to receive an MR image of their knee and to attend the CHESM laboratory for baseline assessment.

C) Participant Incentives:

[Do you propose to reward and/or reimburse and/or compensate participants in any way? If yes, give details here and comment on the special considerations discussed in NS §2.2.10 and NS §2.2.11. If no, state "N/A."]

All participants who are enrolled in the study will receive an Ossür Unloader One© knee brace, to wear during the 8-week study, and to keep once participation is complete.

D) Participant Task(s):

[What will participants be asked to do? What is the approximate time commitment required of each participant? If using records or data only, state "N/A." If your research will be conducted in schools during class time, give details of the alternate activity arranged for students in the class who will not be participating in the research.]

Potentially eligible participants who have not had a radiographic x-ray within the past 12 months and have received their plain language statement and consented will attend one of three listed centres for an x-ray of their knee. For participants with knee osteoarthritis in both knees, the most symptomatic will be studied. If the participant is deemed eligible after screening, they will be invited to participate in the study and undergo baseline assessment and attend The Murdoch Children's Research Institute at The Royal Children's Hospital to undergo MR imaging of their knee. Baseline and follow-up assessment will include completing a questionnaire booklet either online or paper form (attachment 5) and undergo a series of maximal contractions of lower-limb muscles and gait/functional analysis at the CHESM human movement lab (~2 hours). At baseline, gait/functional analysis will be assessed in a randomly selected order for the following four conditions: 1) brace 2) no brace 3) flat shoes and 4) supportive shoes and 5) combined brace/flat shoes. At the 8-week follow-up, participants will complete additional questionnaires and then undergo gait/functional analysis for following two conditions, assessed in a random order: 1) brace; 2) no brace. For each condition at each time- point, 6 trials will be acquired. This process is summarised below in Figure 1.

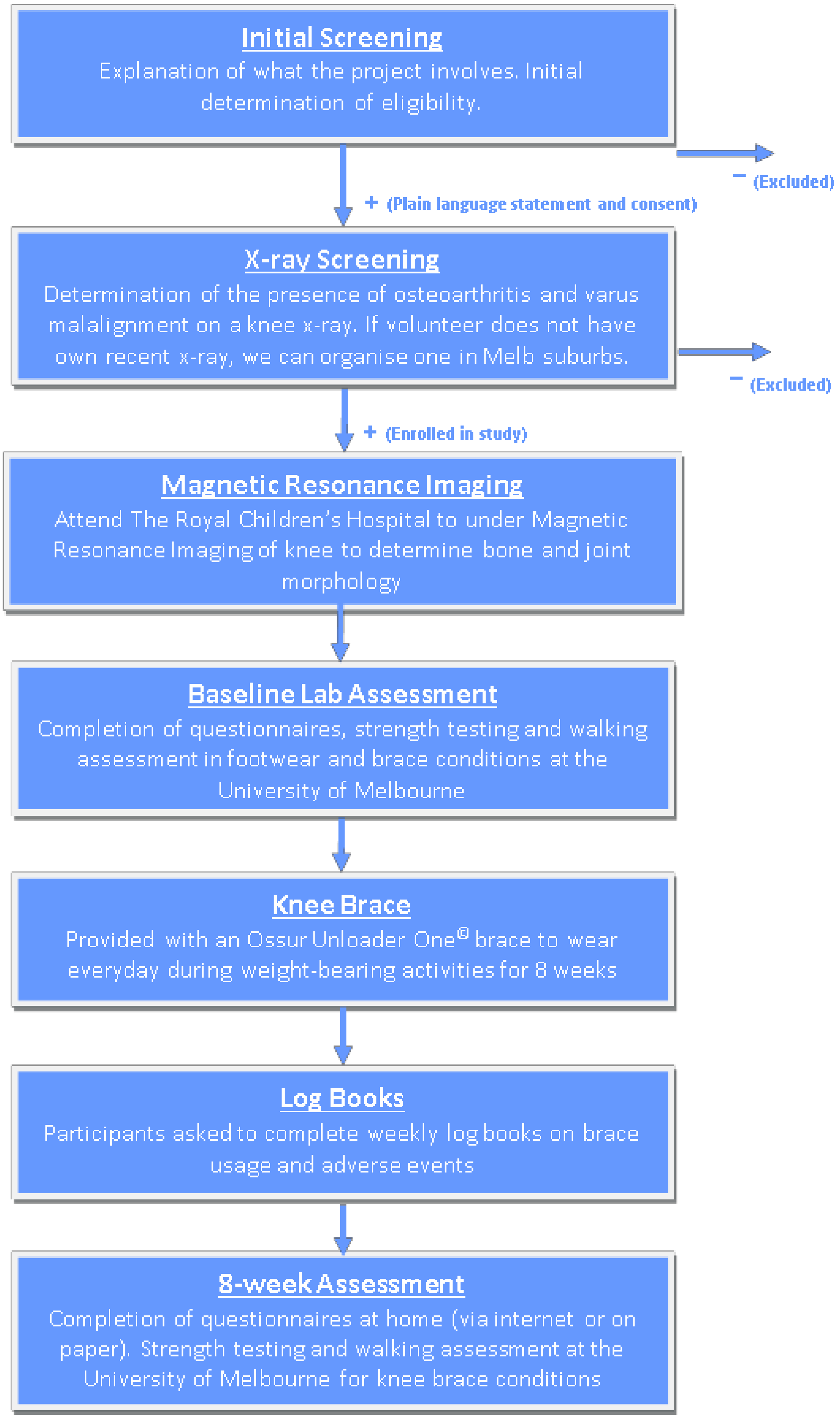

Figure 1. Flowchart outlining study process.

Brace Intervention

Participants will be fitted with an Ossür Unloader One© brace on their affected knee during the baseline assessment by a student investigator trained by the manufacturing company. Participants will be instructed to wear the brace every day during activities (i.e., all weightbearing tasks) and take it off at night. This dosage is based on previous research that have used a similar brace in this population.[4]

E) Data/Material Collection Technique(s):

[What data/materials will be collected? Where will the data be collected? List/describe all sites.]

Baseline

Descriptive data

Variables will be obtained at baseline and will include age, sex, duration of knee osteoarthritis symptoms, severity of knee osteoarthritis and knee alignment on radiographs, treatment expectations, body mass index and medication use. All will be collected by questionnaire (attachment 5) except:

- Radiographic severity of knee OA in tibiofemoral joints: Osteoarthritis will be graded via the Kellgren and Lawrence system[1] (overall grade 0-4). A Bilateral x-ray of the knee will be collected for all participants at baseline at one of: Bridge Road Imaging VIC, Brunswick Diagnostic Imaging VIC, or Blackburn South Radiology VIC.

- Anatomical knee alignment: will be determined from the posterior-anterior x-ray obtained above.

- BMI: height and weight will be measured, and BMI will be computed.

MR Imaging

Bone and joint morphology will be determined based on magnetic resonance imaging. At baseline, MR images of the knee joint will be collected. We intend to use a 3 Tesla magnet (Siemens at Murdoch Children's Research Institute (MCRI), The Royal Children's Hospital) and dedicated knee coil. The MCRI has confirmed the ability to provide these services, however the acquisition of services agreement is awaiting review and appropriate signatures at the MCRI business development and legal office (attachment 6). Images will be taken from the tibial tuberosity to 1cm above the femoral condyles. MR imaging sequences will be set to optimise the visibility of bone-cartilages and ligaments, with short scan times. The following parameters are proposed based on an existing protocol[6] (attachment 6).

Baseline and 8-week follow-up

Gait Analysis

A standard gait analysis protocol, commonly used in clinical and research settings, will be used to collect data at our gait labs (CHESM, University of Melbourne). These data consist of 3D marker trajectories (Vicon MX, Oxford, UK), GRFs (AMTI Inc., Watertown, MA) and EMGs of muscle activation patterns (Noraxon DTS 2400 wireless, AZ, USA). First, skin-surface electrodes will be applied atop the main muscles of the affected lower-limb in order to acquire EMGs. The participant will then be asked to perform a series of maximal muscle contractions*. Subsequently, retro-reflective markers will be applied to the participant's skin surface atop anatomic landmarks. The participant will then be asked to walk at a self-selected speed overground where our systems will collect marker positions, GRFs and EMGs. In a randomly selected order, the participants will then compete walking trials for the following five conditions: 1) brace (Unloader One©, Ossur, Iceland) 2) no brace 3) flat shoes; 4) supportive shoes and 5) combined brace/flat shoes. We will also ask participants to perform functional tasks commonly performed in exercise programs prescribed to people with knee OA. For examples – lunge, squat and step-up. At the 8-week follow-up, the following two conditions will be assessed in a random order: 1) brace; 2) no brace. For each condition at each time-point, 6 walking trials will be acquired.

*Maximum muscle contractions: a hand-held dynamometer will be used to measure maximum isometric strength (peak torque; Nm/kg) of the hip abductors, extensors and flexors.[7] Maximum voluntary isometric torque of the knee extensors and flexors at 60 degrees knee flexion will be measured in sitting using a KinCom isokinetic dynamometer.

Questionnaires:

- *Self-reported knee pain, other symptoms, function in daily living, function in sport and recreation, and knee-related quality of life* over the past week will be measured using the Knee Injury and Osteoarthritis Outcome Score (KOOS).[8] The KOOS contains 42 questions using a 5-point Likert scale from 0 to 4, which is normalised to a score of 100, where a lower score indicates worse symptoms.

- *Knee pain* will also be assessed with a Numeric Rating Scales rating overall knee pain and knee pain during walking[9] in the past week with terminal descriptors of "no pain" (score 0) to "worst pain possible" (score 10).

- *Health-related Quality of Life* will be measured with the Assessment of Quality of Life (AQoL, version II)[10] instrument, which contains 20 items assessing 6 dimensions: Independent living, mental health, relationships, pain, coping and senses. Responses are on a 5-point likert scale.

- *Self-efficacy for pain and function:* The arthritis self-efficacy scale[11] will assess confidence for managing pain (5 questions), symptoms (6 questions) and physical function (9 questions) using a 10-point numeric rating scale (1 = very uncertain to 10 = very certain).

8-week follow-up only

- *Perceived global change in pain, physical function and overall*: will be measured at the 8-week follow-up on a 7-point scale. The terminal descriptors on the 7-point scales will be "much worse" to "much better". Participants who report 'moderately better' or 'much better' will be classified as improved.

- *Co-intervention use:* (e.g. medications and any other treatments specifically for knee pain) will be monitored by a questionnaire at the 8-week follow-up (attachment 5)

Other measures (log books)

Adherence, comfort and confidence:

Daily brace use (in hours and/or minutes) will be self-reported in a log book (attachment 7). After each week, they will also rate their perceived overall level of compliance with daily use on a 11-point numerical rating scale (NRS) (with terminal descriptors of "have not worn brace at all" and "worn brace completely as instructed"). Based on these weekly ratings, the researchers will compute an average time wearing the brace and level of perceived compliance to daily brace use. The log book will be returned to the investigators during the follow-up session or posted at no cost to the participants.

Comfort and confidence

Participants will report weekly comfort levels (11-point scale with terminal descriptors "extremely uncomfortable" to "extremely comfortable")[12] and weekly confidence levels whilst performing daily tasks (11-point scale with terminal descriptors "extremely unconfident" to "extremely confident") within a log book (attachment 7).

Adverse events

Participants will be instructed to report adverse events from using the brace – if any – at the end of each week in a log book (attachment 7).

F) Data Analysis:

[How will data/materials be analysed? What methods/techniques/theories will be used? If qualitative methods will be used, refer to NS §3.1.]

Neuromusculoskeletal Modelling

Kinematic, GRF and EMG data from the walking trials will be organised and processed for use in OpenSim[12] by a software toolbox called MoToNMS[13] within Matlab (Mathworks). We will then integrate our 3D anatomical knee surfaces into our anatomical model[14] by segmenting the bones, cartilage and ligaments in each MRI slice using Mimics (Materialise, Leuven). We will then utilise the 3D marker trajectories to linearly scale anatomical models of each participant within OpenSim and subsequently use inverse kinematics, inverse dynamics and muscle analysis tools to determine the lower-limb joint kinematics, joint moments and muscle tendon unit kinematics. This data will then be used as inputs into a planar mechanism[15] by the Calibrated EMG-Informed Neuromusculoskeletal modelling toolbox (CEINMS)[16] to solve for, and estimate medial knee joint contact force, normalised to bodyweight. This method will also provide data relating to relative muscle and external load contributions to knee joint contact forces, as well as key lower-limb muscle co-contraction values.

Statistics

Descriptive statistics (i.e. means and standard deviations) will be used to summarise participants characteristics and self-reported measures, assess feasibility of the bracing (e.g. participant retention), adherence to the intervention (e.g. self-reported number of hours wearing the brace) and safety of the intervention (e.g. nature and number of adverse effects). To assess the immediate effect of footwear and brace conditions a linear mixed statistical model that includes between condition interactions and random participant effects will be used to assess

differences in medial tibiofemoral joint contact force between: 1) braced and unbraced conditions and 2) stable and flat shoe conditions and 3) combined brace/flat shoes and each intervention in isolation. Paired t-tests will be used to compare baseline and follow-up measures for medial tibiofemoral joint contact force and patient reported outcomes.

Key references:

1. Altman R, et al. (1986). Development of criteria for the classification and reporting of osteoarthritis. Classification of osteoarthritis of the knee. Diagnostic and Therapeutic Criteria Committee of the American Rheumatism Association. *Arthritis and rheumatism*, 29(8), 1039-49.
2. Kellgren JH, et al. (1957). Radiological assessment of osteo-arthrosis. *Annals of the rheumatic diseases*, 16(4), 494-502.
3. Sharma L, et al. (2001). The role of knee alignment in disease progression and functional decline in knee osteoarthritis. *JAMA*, 286, 188–95.
4. Sattari S, et al. (2011). Comparison the effect of 3-point valgus stress knee support and lateral wedge insoles in medial compartment knee osteoarthritis. *Iranian Red Crescent Medical Journal*, 13(9), 624-8.
5. Paterson KL, et al. (2017). Effects of footwear on the knee adduction moment in medial knee osteoarthritis: classification criteria for flat flexible vs stable supportive shoes. *Osteoarthr Cartil,* 25, 234–241.
6. Brito da Luz S, et al. (2017). Feasibility of using MRIs to create subject-specific parallel-mechanism joint models. *Journal of Biomechanics*, 53, 45-55.
7. Pua YH, et al. (2008). Intrarater test-retest reliability of hip range of motion and hip muscle strength measurements in persons with hip osteoarthritis*. Archives of Physical Medicine and Rehabilitation*, 89(6), 1146-•54.
8. Roos EM, et al. (1998). Knee injury and Osteoarthritis Outcome Score (KOOS)-validation of a Swedish version. *Scandinavian Journal of Medicine & Science in Sports*. 8(6):439-48.
9. Bellamy N. (1997). Osteoarthritis clinical trials: candidate variables and clinimetric properties. *J. Rheumatol.,* 24(4):768-778.
10. Osborne RH, et al. (2003). Quality of life assessment in the community-dwelling elderly: validation of the Assessment of Quality of Life (AQoL) Instrument and comparison with the SF-36. *J. Clin. Epidemiol,* 56(2), 138-147.
11. Lorig K, et al. (1989). Development and evaluation of a scale to measure perceived self-efficacy in people with arthritis. *Arthritis Rheum,* 32(1):37-44.
12. Newcomb, N. R. A., et al. (2018). Effects of a hip brace on biomechanics and pain in people with femoroacetabular impingement. *Journal of Science and Medicine in Sport*, 21(2), 111-116.
13. Delp SL, et al. (2007). OpenSim: open-source software to create and analyze dynamic simulations of movement. *IEEE Transactions on Bio-medical Engineering*, 54(11), 1940-50.
14. Mantoan A, et al. (2015). MOtoNMS: A MATLAB toolbox to process motion data for neuromusculoskeletal modelling and simulation. *Source Code for Biology and Medicine*, 10(1):12.
15. Rajagopal A, et al. (2016). Full-Body Musculoskeletal Model for Muscle-Driven Simulation of Human Gait. *IEEE Transactions on Bio-medical Engineering*, 63(10), 2068-79.
16. Winby CR, et al. (2009). Muscle and external load contribution to knee joint contact loads during normal gait. *Journal of Biomechanics*, 42(14), 2294-300.
17. Pizzolato C, et al. (2015). CEINMS: A toolbox to investigate the influence of different neural control solutions on the prediction of muscle excitation and joint moments during dynamic motor tasks. *Journal of Biomechanics*, 48(14), 3929-36.

3. Risks, Benefits and Monitoring

3.1 Potential Risks to Participants	Does your research project pose any potential risks to participants? What are those risks? How will they be negated, minimised or managed?<br>**Refer to *NS* §2.1.**<br>**Note that the risks you identify here should also be described in your Plain Language Statement (PLS). Attach a copy of any distress protocol or adverse event protocol (if applicable).**

A) Potential Risks

[Identify, as far as possible, any potential risks to participants associated with the research project. Risks may arise from the nature of questions that participants are asked (such as discussing sensitive or distressing topics), or the tasks that participants will do, or the procedures that they will undergo. Potential risks might be physical, psychological, emotional, social, legal or economic in nature (this list is not exhaustive). Risks also may be associated with the research setting (e.g. outdoors, in unsecure housing, or in countries other than Australia). If you believe that any potential risks are minimal, please state this and explain why.]

X-ray evaluations

For those participants who have consented and do not have a suitable x-ray within the past 12 months, this study will involve exposure to a small amount of radiation as part of the screening process.

MR imaging

If a participant is deemed eligible, they will undergo MR imaging of the study knee as part of the baseline assessment. However, contraindications do exist for some people for this imaging technique that need to be considered.

Laboratory data collection

The collection of laboratory data is associated with minimal risk to participants. These include fatigue or knee pain due to repeated walking and during the maximal strength testing.

Intervention

There is also a risk that participants may experience increased hip, knee or back pain whilst wearing the knee brace during the 8-week intervention period.

B) Risk Management Strategy

[Describe what measures you have in place to negate, minimise or manage the potential risks you have identified. Depending on the type(s) of risks involved, participants may also need additional support (e.g. external counselling) during or after the study. Attach or include a copy of any distress protocol or adverse event protocol which you havedeveloped.]

X-ray evaluations:
As part of everyday living, everyone is exposed to naturally occurring background radiation and receives a dose of about 2 millisievert (mSv) each year. The additional effective dose patients will receive from entering this trial and require an x-ray is approximately 0.04 mSv. At this dose level, no harmful effects of radiation have been demonstrated as any effect is too small to measure (attachment 8). Studies suggest any risk is minimal. To minimize radiation exposure, we will not expose those who have had a recent knee x-ray (within the past 12 months) to a new x-ray.

MR Imaging:
If operator and safety procedures as well as eligibility requirements are met, MR imaging is considered a biologically safe imaging technique. As part of the induction procedures, participants will be questioned for contraindications to MR imaging. Participants will be screened over the telephone and excluded if absolute contra-indications are present (Attachment 3). They will also undergo further screening at Medical Imaging Department at the Royal Children's Hospital, where they will complete a standardised MRI form used prior to scanning any patients. Along with MRI appointment details, participants will receive an MRI information leaflet and a reminder of requirements of safe imaging procedures (ie removal of eg belts, jewellery, watches, coins, etc.) (Attachment 4). During the MR imaging procedures, participants will be given headphones for the noise that occurs during MR imaging.

Laboratory data collection
The risks associated with maximal strength and gait/functional testing in the laboratory will be minimised by asking the participant regularly about any pain and informing them that they are to let the tester (student researcher) know if

during testing. We also give regular rest periods. The research student conducting the testing has qualifications in physiotherapy and is experienced in the clinical testing and management of people with knee osteoarthritis.

Intervention

Whilst wearing the knee brace (baseline to 8 weeks), there is a risk that participants may experience an exacerbation of knee pain or compensatory hip or back pain as a result of wearing the brace. To minimise this, all participants will be shown how to correctly fit their brace by the student researcher who has been trained by the manufacturing company, and will fit the brace in accordance with the manufacturer's specifications. The participants will also be given the Investigator's phone details and asked to contact the team for advice if they suffer any adverse effects from wearing the brace. If necessary, participants will not be withheld from additional co-interventions. Additionally, any lower limb condition other than knee osteoarthritis that would interfere with appropriate use of the brace will be excluded during screening.

**3.2 Potential Risks to Non-Participants**	Does your research project pose any potential risks to non-participants? (This could possibly include risks to researchers or independent contractors.) If so, how will these risks be minimised? **Refer to *NS* §2.1.**

[Describe any potential risks and your risk management strategy for non-•participants, if applicable. Risks to non-•participants might include things such as potential breach of privacy, stigmatisation of a particular group, or knowledge about familial genetics. If you believe that any potential risks to non-•participants are minimal, please state this and explain why.]

No

**3.3 Risks, Benefits and Justification**	In light of the risks and expected benefits of the research project, explain how the expected benefits of the research justify any risks it may pose. **Refer to *NS* §1.6 - §1.7 and *NS* §2.1.**

A) Expected Benefits

[Describe any expected benefits of this research. Include potential benefits to the community or society, and any specific potential benefits to participants, beyond general positive feelings that may arise from participating in research and having one's voice heard. Note that it is generally not necessary to demonstrate specific benefit to participants in order to show that research is ethically justifiable.]

This research will provide insight into the effects of immediate and short-term use of valgus knee bracing, and immediate effects of two different over-the-counter footwear types with regards to knee joint loading, which is a potential predictor osteoarthritis progression. Overall, understanding whether and in what way people modify knee loading patterns with the brace or certain footwear types will inform future studies. Findings will inform clinicians whether a knee brace, footwear or combination of both is best at reducing harmful knee loads and will thus assist clinical decision-making and ultimately benefit patients with knee osteoarthritis.

All study participants will receive an Ossur Unloader One© brace to keep at the end of the study. The Unloader brace is expected to provide pain relief and improve physical function, as demonstrated by previous research.

B) Justification of Risks by Expected Benefits

[Explain how the expected benefits of the research justify the risk(s) which you identified in questions 3.1 and 3.2. Pay particular attention to any risk(s) to participants that are greater than inconvenience.]

The outcomes of this study will provide an in depth understanding of how contact forces within our knee joint responds to bracing and footwear types. Ultimately, the project has the potential to influence contemporary choice of footwear and braces in people with osteoarthritis and reduce the personal and societal burden of this increasing public health problem. The risks associated with this study are relatively small and are thus outweighed the anticipated benefits of this study.

3.4 Management and Monitoring	How will researchers manage and monitor conduct of the research project? **Refer to** ***NS* §5.5**.

A) Management

[Provide details of how and by whom the research project will be managed, throughout the life of the project, to ensure that it complies with the protocols set out in this application, and with all relevant legislation and regulations. Address cases where several people are or may be involved in recruiting, interviewing, obtaining data or data analysis.]

The responsible researcher, Dr Michelle Hall, will have overall responsibility for the conduct of the project. Regular meeting, typically weekly, will be scheduled between the co-investigators. At these meetings, the student investigator will provide an update on the status of the project, outcome of the data collection; any issues that may arise will be discussed. Further to this, any discussion of data will be on anonymised data only.

B) Monitoring

[If the research will be carried out at some distance from the responsible researcher (i.e. interstate or in countries other than Australia), describe the systems in place to ensure compliance with the research protocols you have outlined in this application. If the research will be undertaken by a student, describe how the student will be supervised to ensure compliance with the protocols, including details of any local supervision to be organised for research conducted overseas or interstate.]

The responsible researcher, Dr Michelle Hall, will be responsible for ensuring that this project is conducted in accordance with University's human research ethics guidelines, the national Statement on Ethical Conduct in Human Research, and that the project adheres to the University's HREC protocols.

C) Independent Contractors

[If any independent contractors (i.e. persons not listed in Themis as researchers on this project) will be carrying out any part of the research, provide details of the contractors involved, explaining their role and their qualifications/experience to fulfil this role. Include details of any training that will be provided to the contractors. Confirm that the contractors will be provided with a copy of the approved ethics protocol,and advised of their responsibilities in relation to the research. If no independent contractors will be involved, state "N/A".]

<u>X-ray evaluations:</u>

A radiographer and radiologist will be performing the x-ray evaluations at the Epworth Hospital Richmond, Blackburn South Radiology and Brunswick Diagnostic Imaging. These x-rays will be paid for out of project funds. The Principal Investigator will provide a copy of the approved ethics protocol to each Radiology Department and staff, in addition to informing the radiologist/s of their responsibilities. These Radiology Departments have performed the x-ray evaluations for several of our previous projects using the same imaging protocol (e.g. HREC 1239045.1).

<u>MR imaging- acquisitions:</u>

A radiographer and radiologist will be performing the MR imaging evaluations at the Murdoch Children's Research Institute within The Royal Children's Hospital. These MR images will be paid for out of project funds. The Principal Investigator will provide a copy of the approved ethics protocol to the relevant clinics and staff, in addition to informing them of their responsibilities. These Radiology Departments have performed the MR imaging evaluations for our previous projects (e.g. HREC 1442016.1).

Dr Michelle Hall will oversee all aspects of the study.

4. Consent

4.1 Obtaining Informed Consent	**Type an "X" in the left-hand column beside as many of the following options as apply** to your research project. **Use the space provided below** to explain how you will obtain informed consent from participants. If you seek a waiver of consent, or the use of opt-out consent, use the space provided to justify your request. **Refer to *NS* §2.2, *NS* §2.3**.

X	**Written consent** will be sought from (or on behalf of) participants.	→ **Refer to *NS* §2.2.6.**<br>→ **Attach a copy of your consent form(s).**

	**Verbal consent** will be sought from (or on behalf of) participants.	→ **Refer to [NS §2.2.5 - §2.2.6](#).**<br>→ **Explain why you have chosen this form of consent, and how an individual's consent to participate will be recorded.**<br>→ **Attach a copy of your consent script(s).**
	**Consent will be implied,** rather than explicitly obtained.	→ **Refer to [NS §2.2.5 - §2.2.6](#).**<br>→ **Explain why you have chosen this form of consent.**
	**Third parties (e.g. parents/guardians of minors) will provide consent** on behalf of participants.	→ **Refer to [NS §2.2.12.](#)**<br>→ **Explain who will be providing consent on behalf of participants and why.**
	**Third parties (e.g. community elders, school boards) will be involved** in whole of community participation decisions.	→ **Refer to [NS §2.2.13](#).**<br>→ **Provide details of which third parties will be involved, why they will be involved, and how this will be accomplished.**
	This application seeks a **waiver of consent.**	→ **Explain why you are seeking this option. Justify your request by referring to the conditions described in [NS §2.3.10 - §2.3.11](#).**
	This application proposes to use **opt-out consent.**	→ **Explain why you are seeking this option. Justify your request by referring to the conditions described in [NS §2.3.6](#).**

[Write your responses here. If you will be obtaining consent from participants, describe who will obtain consent. Explain how it will be established that potential participants are competent to understand the research and to participate voluntarily, particularly if they are in a dependent relationship with the researcher(s). If you will not be obtaining consent from individual participants, justify your request for a waiver of consent, or for use of opt-•out consent.]

**4.2 Limited Disclosure**	Do you propose to use limited disclosure, concealment or deception for this research project? (Answer Yes or No. If Yes, use the space below to explain.) **Refer to [NS §2.3](#).**	
	**YES or NO:**	**NO**

*[If NO, you may leave this space blank. If YES, provide a justification for the limited disclosure, concealment or deception. Comment on the special considerations discussed in **NS §2.3**. Indicate whether you intend to debrief participants, and justify that position. If you are seeking a waiver of consent for all participants, select NO.]*

**4.3 Future Use of Data, Materials, or Tissues**		Do you intend for the data and/or materials and/or tissues collected for this research project to be reused in future research? **Type an "X" in the left-hand column beside as manyof the following options as apply** to your research. **Use the space provided** to specify which data/materials/tissues will be reused, if any. **Refer to [NS §2.2.14](#)** and **[NS §3.1](#)**
	Consent will be **specific.**	→ Data/materials/tissues will be used *only* for this research project (i.e. **no future use**).
**X**	Consent will be **extended.**	→ Data/materials/tissues used in this research project may also be used in future projects that are *closely related* to this project, *or in the same general area* of research as this project. Make this clear in PLS
	Consent will be **unspecified.**	→ Data/materials/tissues used in this project may also be used in *any* future research. Make this clear in PLS

[If data/materials/tissues from this research project will not be reused, select "specific" above and state "N/A" here. If data/materials/tissues will be reused, describe which of them will be reused explain and how such future use will occur. If different conditions of consent apply to different data/materials/tissues, explain which conditions apply to which data/materials/tissues. If you will also be completing the Privacy and Databanks Module, you may simply write "Refer to Privacy and Databanks Module" here.]

Radiographic x-rays may be used for future research within the acceptable duration of recent x-rays to avoid unnecessary exposure to ionising radiation of eligible participants.

4.4 Conflict of Interest	Does your research present or involve any conflict of interest, whether potential, real, or perceived; or will the researcher(s) have dual roles in relation to the participants? (Answer Yes or No. If Yes, use the space below to explain.) **Refer to *NS §5.4*, University of Melbourne Research Integrity and Misconduct Policy (MPF1318), and *Australian Code for the Responsible Conduct of Research §7.2*.**	
	**YES or NO:**	**YES**

This study is in part sponsored by an industry partner, Ossur. However, these study sponsors will not play any role in the collection, analysis or interpretation of data, writing of the manuscript, or decision to submit the manuscript for publication.

4.5 Information for Participants	How will relevant information about the research project be provided to potential participants? **Attach a copy of any advertisement (print or online), Plain Language Statement (PLS), consent form, letter, email, telephone script, and/or debriefing statement** to be used. **Refer to NS §5.2.25** and **NS 3.1**

[Explain how participants will be informed about the research project. If applicable, explain what arrangements will be made forinforming participants with low literacy skills, and/or for translation/interpreting of these materials for participants who are speakers of languages other than English. If you are seeking a waiver of consent for all participants, state "N/A."]

Participants will be provided with an information letter and a Plain Language Statement (attachment 4).

Plain Language Statement (PLS): Your PLS must satisfy the requirements set out in the *National Statement* (***NS* §2.2.1 - §2.2.3, §2.2.6**). The Research Ethics and Integrity's website has **guidance on composing your plain language statement**, as well as an **example PLS template**. A list of PLS requirements is also provided at the end of this form. ***Ensure that your PLS is written in plain language. Ensure that the information contained in your PLS is consistent with the information in your application.***

Consent Form: Your consent form must satisfy the requirements set out in the *National Statement* (***NS* §2.2**). The Research Ethics and Integrity's website has **guidance on composing your consent form**, as well as an **example consent form**. A list of consent form requirements is also provided at the end of this form. ***Ensure that your consent form is written in plain language. Ensure that the information contained in your consent form is consistent with the information in your application.***

5. Dissemination and Data Management

5.1 Providing Results to Participants	How will the results of the research project be provided to participants in an accessible format? **Refer to *NS* §1.5 and NS 3.1**

[Describe how participants will be given access to the results of the research. If you will only be using pre-•collected data and/or tissue, state "N/A". If you are seeking a waiver of consent, state "N/A".]

Participants will be offered the opportunity to receive a lay-written summary of the study findings by ticking the appropriate box on the consent form. The report contents will remain unrelated to individual responses to maintain anonymity of participants.

5.2 Reporting Project Outcomes	How will outcomes of the research project be made public? **Refer to *NS* §1.3 and NS 3.1**

[Describe the format and means by which you intend to make the project's results public.]

Findings will be presented at conferences and published in peer-reviewed journals.

5.3 Data Management	How do you propose to manage the data collected in this research project? Specify what types of data will be collected, how they will be stored and in what format. How will access to the data be controlled and by whom? Discuss retention, security, and data sharing plans. What measures will be taken to protect participants' privacy, and their data?<br><br>**Refer to *NS* §1.11, NS 3.1, the *Australian Code for Responsible Conduct of Research §2*, and the University of Melbourne Research Integrity and Misconduct Policy (MPF1318).**

A) Privacy and Confidentiality

[What measures will be taken to protect participants' privacy and the confidentiality of participants' data? Describe the format in which the data will be stored (e.g. digital video file, database of survey responses, paper forms.) Describe whether the data will be identifiable. That is, will it be possible for researchers or others to match data to specific participants? If so, how will this be possible? If not, how will such matching be prevented?]

All participant data will be de-identified and stored using a code. Other hardcopy information, codes and all identifying information will be kept in separate locked filing cabinets. Electronic data will be stored on password-protected folders.

B) Security and Storage of Data

[What short-•term storage will you use during the data collection phase? Whose responsibility will it be to manage this? What long-•term storage will you use after the data collection phase? Whose responsibility will it be to manage this? Who will have access to unprocessed (raw) data? What security measures will be in place to control access to data?
NOTE: If your research will generate digital and non-•digital data, separate this section into two parts: "Security and Storage of Non--Digital Data" and "Security and Storage of Digital Data."]

Identifiable data:

For participants who are recruited through the community, initial online screening information will be stored within a website (SurveyGizmo) and accessible only by password to the researchers. Phone screening forms for all potentially eligible participants will be completed on paper by the student investigator and stored in a locked filing cabinet accessible only to the researchers. Details of people screened will be stored electronically, accessible only to the researchers stored securely on password-protected servers. Consent forms will be completed on paper. Paper copies will be stored with other identifiable data in a locked filing cabinet. Electronic data will be stored electronically (e.g. Microsoft Excel) and accessible only by password to the researchers.

For those participants who undergo a radiographic x-ray, images will be stored electronically at the radiology clinics involved in the study and securely stored and subject to the normal confidentiality guidelines adhered to at each clinic. Researchers will access the x-ray images via log-in to the clinic software and will export de-identified images, label with appropriate subject codes and store securely on password-protected servers. We will not retain x-ray images of the participants who present with their own eligible x-rays- these will be posted back to participants after radiographic severity and alignment has been graded (recorded on paper and stored in locked filing cabinets). For the x-ray screening assessments, Prof. Hinman will complete a screening form in surveygizmo/REDCap accessible only to her and the other researchers by password. Data from eligible participants will be exported to Microsoft Excel and stored securely on password-protected servers.

MR images will be stored electronically at the radiology clinic (The Murdoch Children's Research Institute, The Royal Children's Hospital) involved in the study and securely stored and subject to the normal confidentiality guidelines adhered to by this clinic. Researchers will access the MR images via log-in to the clinic software and will export de-identified images, label with appropriate subject codes and store securely on password-protected servers. Data will be exported by researchers for analysis within the software program Mimics (Materialise, Leuven) and stored securely on password-protected severs.

Re-identifiable data:

Questionnaires may be completed on paper or electronically, and will contain only subject codes, and no identifying information. Paper copies will be stored in locked filing cabinets, separate from a cabinet containing any identifiable data and only accessible to the researchers. Electronic copies will be stored in the SurveyGizmo website, accessible only to the researchers by password protection. Data from within SurveyGizmo will eventually be exported to Microsoft Excel and other statistical packages used by the researchers for analyses and stored securely on password-protected servers.

C) Retention

[For how long will you keep the data generated by this research project? How will you ensure that data is retained if/when the researcher(s) leave the University? For data that are not intended to be kept indefinitely, how will you eventually dispose of the data?
*NOTE: the minimum retention period for research data and primary materials is five years after the last publication, or public release, arising from the research (**University Code §2.1**). Longer minimum retention periods apply for certain types of research – refer to the requirements of relevant regulations.]*

Data will be disposed of by the Responsible Researcher or one of the c-researchers 15 years after publication. Ultimate disposal of paper records will be via shredding, digital records permanently deleted and audio tapes wiped or ruined.

6. Other Issues

6.1 Other Ethical Issues	Are there any other issues, not addressed above or in additional modules, which are relevant to the ethical review of your research project? **Refer to the relevant sections of the *National Statement* identified in the Specific Guidelines Checklist, if applicable.**
[Use this space to address any relevant ethical issues that are not addressed elsewhere in this application. If there are no other issues relevant to the ethical review of your research project, state "N/A."]<br>N/A	

Attachments Checklist	
Review your answers above to determine which attachments (if any) are required for your application. **Type an "X" in the left-hand column beside all items that apply to your research project.** Attach a copy of the items you have selected.	
X	**Plain Language Statement (PLS) for Participants**
X	**Consent Form for Participants**
	**Additional PLS(s)** (e.g. for parents, teachers, schools)
	**Additional Consent Form(s)** (e.g. for parents, teachers, schools; or assent forms for children)
X	**Recruitment Materials** (e.g. advertisement(s), posters, letter(s) or email(s) of invitation)
X	**Questionnaire(s) and/or Survey Instrument(s)**
	**Measure(s) and/or Scale(s)**
	**List of Interview Questions and/or Themes**
	**List of Focus Group Questions and/or Themes**
	**Participant Distress Protocol**
	**Adverse Event Protocol**
	**Debriefing Statement**
	**Approval(s) of research by an HREC external to the University of Melbourne**
	**Other External Approval(s)** (e.g. schools, communities)
	**Full Protocol** (for Medical Research)
	**Translations and/or Back-Translations** (where languages other than English used)
	**Privacy and Databanks Module**
	**Body Tissue and Genetic Research Module**
X	**Ionising Radiation Module**
X	**Interventions, Therapies and Trials Module**
X	**Other Documents** (e.g. contracts, agreements) – specify which:
	In-progress acquisition of services agreement and confirmation letter – The Murdoch Children's Research, The Royal Children's hospital Royal Children's Hospital

Plain Language Statement (PLS) Requirements:

1. Clearly identify the University of Melbourne (i.e. by prominent placement of the University's logo) and the department(s)/ school(s)/faculty(-ies) involved. If printed, the PLS should be on University of Melbourne letterhead.
2. Clearly identify the title of the project, and the name(s) and contact details of the Principal Researcher and Other Researchers. For student projects, specify the student's level of study.
3. Clearly explain the purpose of the research project.
4. Clearly explain what participants will be asked to do, and provide an estimated time commitment.
5. If participants will be photographed, audio- or video-recorded, clearly state as much.
6. Clearly explain any risks arising from participation, as well as any procedures or measures in place to minimise such risks.
7. Describe any expected benefits to the wider community. If applicable, also describe any expected benefits to participants.
8. List any payments, incentives or reimbursements to be made to participants.
9. State that involvement in the project is voluntary and that participants are free to withdraw from participation at any time. Explain any implications of withdrawal, including whether it will be possible for participants to withdraw any data already collected from or about them.
10. Describe the likelihood and form of dissemination of the research results, including publication.
11. Describe the arrangements in place to protect the confidentiality of participants' data, and advise participants of any legal limitations to such confidentiality. If the sample size for the project is small, advise participants that this may make them identifiable.
12. The project HREC number (which is the ethics ID number assigned by Themis) and the date and version number of the PLS must appear on the PLS. If the PLS is printed, put this information in the footer.
13. Explain what will happen to participants' data after the research project ends (i.e. how long it will be retained, whether it might be used again for future research and if so who would have access.)
14. Include the following statement: "This research project has been approved by the Human Research Ethics Committee of The University of Melbourne. If you have any concerns or complaints about the conduct of this research project, which you do not wish to discuss with the research team, you should contact the Manager, Human Research Ethics, Research Ethics and Integrity, University of Melbourne, VIC 3010. Tel: +61 3 8344 2073 or Email: humanethics-complaints@unimelb.edu.au All complaints will be treated confidentially. In any correspondence please provide the name of the research team or the name or ethics ID number of the research project."
15. If the research is externally funded, state the amount(s) and source(s) of funding for the research.
16. If there are any potential conflicts of interest for any of the researchers, sponsors (if applicable) or institutions, disclose these potential conflicts of interest.
17. If any participants will be in a dependent relationship with any of the researchers, state that decisions about participation will not affect the dependent relationship. (E.g. students' grades will not be affected if they decline to participate or withdraw from the project at any stage).

Consent Form Requirements:

1. Clearly identify the University of Melbourne (i.e. by prominent placement of the University's logo) and the department(s)/ school(s)/faculty(-ies) involved. If printed, the consent form should be on University of Melbourne letterhead.
2. Clearly identify the title of the project, the name(s) and contact details of the Principal Researcher and Other Researchers. For student projects, specify the student's level of study.
3. If participants will be photographed, audio- or video-recorded, clearly state as much.
4. State that involvement in the project is voluntary and that participants are free to withdraw consent at any time, and to withdraw any unprocessed data previously supplied. Also state that the purpose of the project is research.
5. Describe the arrangements in place to protect the confidentiality of participants' data, and advise participants of any legal limitations to such confidentiality. If the sample size for the project is small, advise participants that this may make them identifiable.

Declaration by the Responsible Researcher

The information contained in this application is, to the best of my knowledge and belief, accurate.

I have read the University's current human ethics guidelines. I accept responsibility for the conduct of the procedures set out in the attached application in accordance with: those guidelines, with the ***University's Research Integrity and Misconduct Policy (MPF1318)***, and with any other condition laid down by the University of Melbourne's Central Human Research Ethics Committee (CHREC), its Human Ethics Sub-Committees (HESCs), or by the Human Ethics Advisory Group (HEAG) which will review this application. I have attempted to identify all risks related to the research that may arise in conducting this research. I acknowledge our obligations as researchers and the rights of the participants stipulated in the ***National Statement on Ethical Conduct in Human Research (2007) - Updated 2018***. I certify that the research team has the appropriate qualifications, experience and facilities to conduct the research described in the attached application, and to deal with any emergencies and contingencies related to research that may arise throughout the life of the project.

If approval is granted, the project will be undertaken in strict accordance with the approved protocol and relevant laws, reg ulations and guidelines.

I, the Responsible Researcher, agree to:

- start this research project only after obtaining final approval from the HESC (if this is a standard project), or the HEAG (if this is a minimal risk project);
- carry out this research only where adequate funding is available to enable the research to be carried out according to good research practice and in an ethical manner;
- provide additional information as requested by the CHREC, HESC, or HEAG;
- provide progress reports to the CHREC, HESC, or HEAG as requested, including annual and final reports;
- maintain the confidentiality of all data collected from, or about, research participants and maintain security procedures for the protection of their privacy;
- submit an amendment if any modification to the research design or protocol is proposed (including any change of researchers) and to proceed with the research only after the amendment has been approved by the HESC (if this is a standard project) or by the HEAG (if this is a minimal risk project);
- notify the HESC (if this is a standard project) or the HEAG (if this is a minimal risk project) in writing immediately if any adverse event occurs during the course of the research;
- notify the HESC (if this is a standard project) or the HEAG (if this is a minimal risk project) in writing immediately if any complaints are received about the research;
- comply with an audit of the research undertaken, if requested by the CHREC, HESC, or HEAG;
- use only the data/tissue samples collected for this research, and for which HESC/HEAG approval has been given.

I certify that all members of the research team have read this application and the ***National Statement on Ethical Conduct in Human Research (2007) - Updated 2018*** and that they have agreed to comply with the provisions of the latter.

Responsible Researcher Name	Signature	Date
Dr Michelle Hall		2/01/2019

Declaration by Human Ethics Advisory Group (HEAG)

For HEAG use only.<br>Enter the date the application was received, then type an "X" in the left-hand column beside each item as applicable.		
**Date Application Received:**		
	**Technical review** has been completed by the HEAG.	The merit of the proposed research project set out in this application has been reviewed on technical grounds.<br>**Refer to *NS §1.1*.**
	**Ethical review** has been completed by the HEAG.	The HEAG has reviewed the proposed research project set out in this application for compliance with the principles of Human Research Ethics.
	The **Minimal Risk** review process is appropriate for the proposed research project set out in this application.	→ **Complete Declaration A (below)**
	The **Standard Project** review process is appropriate for the proposed research project set out in this application	→ **Complete Declaration B (below)**

**Declaration A (Minimal Risk):**	The HEAG has reviewed this project. The HEAG considers the methodological/technical and ethical aspects of the proposal to be appropriate to the tasks proposed. **The HEAG grants approval for this research project to commence.** The HEAG considers that the researcher(s) has/have the necessary qualifications, experience and facilities to conduct the research described in the attached application in a manner that complies with the University's policy on the management of research data and records, and to deal with any emergencies and contingencies that may arise. *[Note: If the HEAG Chair is also a researcher in this project, the declaration should be signed by another authorised member of the HEAG.]*	
**Name of HEAG Chair/Authorised Member**	**Signature**	**Date**

**Declaration B (Standard Project):**	The HEAG has reviewed this project and considers the methodological/technical and ethical aspects of the proposal to be appropriate to the tasks proposed. **The HEAG regards this project as ready to submit to the HESC.** The HEAG considers that the researcher(s) has/have the necessary qualifications, experience and facilities to conduct the research described in the attached application in a manner that complies with the University's policy on the management of research data and records, and to deal with any emergencies and contingencies that may arise. *[Note: If the HEAG Chair is also a researcher in this project, the declaration should be signed by another authorised member of the HEAG.]*	
**Name of HEAG Chair/Authorised Member**	**Signature**	**Date**
Associate Professor Adam Bryant		11/02/2019
