# Supplementary material for: Effect of a valgus brace on medial tibiofemoral joint contact force in knee osteoarthritis with varus malalignment: A within-participant cross-over randomised study with an uncontrolled observational longitudinal follow-up
Source: PLoS One. 2022 Jun 3;17(6):e0257171. doi: 10.1371/journal.pone.0257171 (PMC9165832; doi:10.1371/journal.pone.0257171)
Supplement: S1 Fig — Outcomes and time-points of assessment according to each study aim. (PDF) [file pone.0257171.s001.pdf]

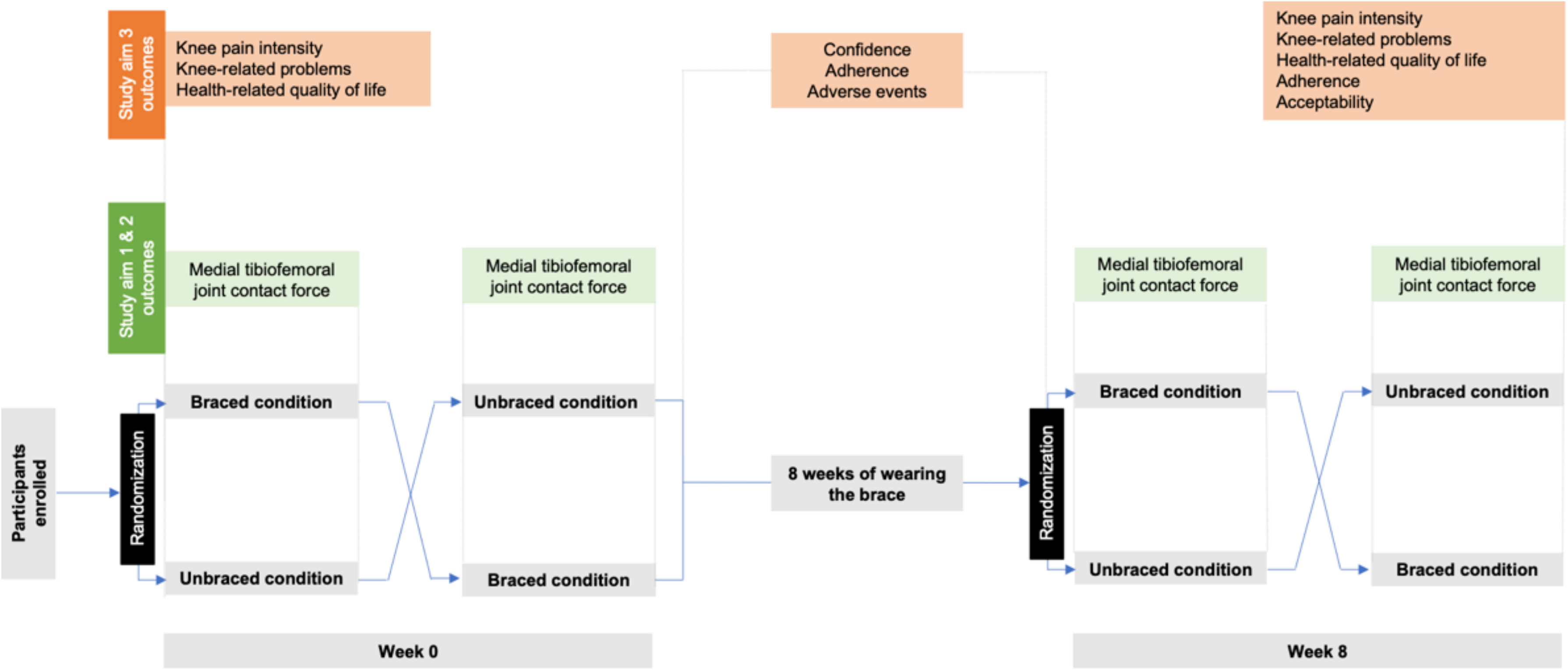
Study aim 3 outcomes
Knee pain intensity
Knee-related problems
Health-related quality of life
Confidence
Adherence
Adverse events
Knee pain intensity
Knee-related problems
Health-related quality of life
Adherence
Acceptability
Study aim 1 & 2 outcomes
Medial tibiofemoral joint contact force
Medial tibiofemoral joint contact force
Medial tibiofemoral joint contact force
Medial tibiofemoral joint contact force
Participants enrolled
Randomization
Braced condition
Unbraced condition
Unbraced condition
Braced condition
8 weeks of wearing the brace
Randomization
Braced condition
Unbraced condition
Unbraced condition
Braced condition
Week 0
Week 8
